# Towards joint *in situ* determination of pressure and temperature in the large volume press exclusively from X-ray diffraction

**DOI:** 10.1107/S1600577523004538

**Published:** 2023-06-21

**Authors:** Robert Farla

**Affiliations:** a Deutsches Elektronen-Synchrotron DESY, Notkestr. 85, 22607 Hamburg, Germany; University of Malaga, Spain

**Keywords:** equations of state, X-ray diffraction, large volume press, high pressure, resistive heating

## Abstract

Since high-pressure devices have been used at synchrotron facilities, accurate determination of pressure and temperature in the sample has been a crucial objective, particularly for experiments that simulate the Earth’s interior. However, in some cases using a thermocouple may have a high likelihood of failure or is incompatible with a high-pressure assembly. To address these challenges and similar issues, we aim to expand a previously proposed solution: to jointly estimate pressure and temperature (*PT*) through *in situ* X-ray diffraction, to cover a wider range of internal *PT* calibrants tested over larger *PT* ranges. A modifiable Python-based software is offered to quickly obtain results. To achieve these aims, *in situ* large volume press experiments are performed on pellets of intimately mixed powders of a halide (NaCl, KCl, KBr, CsCl) or MgO and a metal (Pt, Re, Mo, W, Ni) in the pressure range 3–11 GPa and temperature range 300–1800 K. Although the pressure range was chosen for practical reasons, it also covers an equally important depth range in the Earth (down to 350 km) for geoscience studies. A thermocouple was used to validate the *PT* conditions in the cell assemblies. The key results show that choosing the appropriate calibrant materials and using a joint *PT* estimation can yield surprisingly small uncertainties (*i.e.* <±0.1 GPa and <±50 K). This development is expected to benefit current and future research at extreme conditions, as other materials with high compressibility or high thermal pressure, stable over large *PT* ranges, may be discovered and used as *PT* calibrants.

## Abstract 2

Research in high-pressure devices, such as the diamond anvil cell and the large volume press, requires knowledge of the pressure and temperature in the sample. Here, a large volume press and an internal resistive heater were used to generate high load and heat to various combinations of intimately mixed powders of materials. X-ray diffraction and custom software were used to jointly estimate the pressures and temperatures in the samples and establish calibrants for *in situ* experiments at extreme conditions.

## 1. Introduction

Experiments at high pressures and temperatures (HPHT) require precise knowledge of the conditions inside high-pressure devices, such as the diamond anvil cell (DAC) and the large volume press (LVP), particularly in geosciences where the interior of the Earth must be accurately reproduced (Chanyshev *et al.*, 2022 ▸; Marquardt & Thomson, 2020 ▸; Ishii *et al.*, 2019 ▸; Yu *et al.*, 2019 ▸; Liebermann, 2011 ▸; Murakami *et al.*, 2012 ▸). Today, *in situ* X-ray diffraction (XRD) is commonly used as the best way to obtain pressure information from materials from their calibrated equations of state. With the establishment of high-pressure research at synchrotron facilities and the high output of experimental data using energy-dispersive (ED) and angle-dispersive (AD) XRD in DACs and LVPs, calls are increasing for the implementation of reliable and practical (absolute) pressure scales. The reason for this is that newly published pressure standards of materials show discrepancies in predicted pressures from previous work (Fei *et al.*, 2007 ▸). Several studies have therefore attempted to create self-consistent pressure scales and unified analyses (Fei *et al.*, 2007 ▸; Dewaele *et al.*, 2008 ▸; Sokolova *et al.*, 2013 ▸) and are establishing a new international ruby pressure scale (Shen *et al.*, 2020 ▸). Further complementary measurements of pressure that are not dependent on X-rays for their determination are possible in the DAC, including Brillouin scattering techniques (Speziale *et al.*, 2014 ▸) and ultrasonic interferometry (UI), the latter also possible in the LVP (Li & Liebermann, 2014 ▸; Matsui *et al.*, 2012 ▸; Wang *et al.*, 2009 ▸; Jacobsen *et al.*, 2002 ▸).

Equations of state can take many forms, some semi-empirical, others based on theory. The most widely recognized equation of state (EoS) is the ideal gas law, *PV* = *nRT*. For a comprehensive overview of the different formalisms of equations of state for solids, we refer the reader elsewhere (Angel *et al.*, 2018 ▸; Stacey, 2005 ▸; Holzapfel, 2001 ▸; Anderson, 1995 ▸). In brief, equations of state commonly applied to the Earth’s interior include the Birch–Murnaghan (BM) (Birch, 1947 ▸) and Vinet (Vinet *et al.*, 1987 ▸) equations, which are typically combined with the thermal expansion coefficients of the material to describe thermal pressure. The Mie–Grüneisen–Debye equation of state (MGD) incorporates the thermodynamic definitions of the Debye and Grüneisen parameters (Lemons & Lund, 1999 ▸). The latter is commonly used for extensive datasets covering large pressure–temperature (*PT*) ranges, but users should be aware of the limitations of this model and others (Angel *et al.*, 2019 ▸).

Several specific compounds, such as NaCl, MgO and CaF_2_, have been used for a long time to calibrate pressure (and temperature) in high-pressure devices, such as in early DACs (Hazen & Finger, 1981 ▸). Pioneering work by Zhao *et al.* (1997 ▸) on hBN + NaCl, followed by Crichton & Mezouar (2002 ▸) on Au + NaCl, show promise in the idea of using two materials for joint calibration of pressure and temperature by *in situ* XRD, particularly in the LVP. To further explore this concept, we conducted experiments on a diverse selection of materials and developed software that enables rapid estimation of pressure and temperature within the HPHT cell while using various combinations of these materials. Suitable properties include high compressibility (small isothermal bulk modulus *K*_*T*_, with a small derivative *K*′), high thermal pressure (α*K*_*T*_, where α is the volume thermal expansion), stability and unreactive behaviour over large *PT* ranges, and a simple crystal structure (*e.g.* cubic), offering strong reflections. In particular, (i) α*K*_*T*_ is very useful, because one can expect to combine materials with a low value and a high value reasonably well so that the isochors of the two materials cross at a high angle in *PT* space, and (ii) a cubic symmetry ensures that the deviatoric stress does not change the lattice volume on average, and thus, for the ideal case using angle-dispersive XRD, the measurement of volume returns, via the EoS, the mean stress.

This study explores promising materials with established equations of state, including NaCl, CsCl, KCl, KBr, MgO, Pt, Ni, Mo, W and Re. Gold was not utilized due to its high absorption and low melting point at lower pressures, which made it unsuitable for experiments at the P61B station, where the high-power wiggler beam of PETRA III is used. The objective of this study is to evaluate combinations of materials that provide the most consistent pressure and temperature determination from the corresponding X-ray signal among the samples in the cell assembly. Published equations of state for materials used in this experimental study (and in the software) were selected based on criteria, where applicable, such as calibrations of an EoS of a material combined with other previously calibrated materials (self-consistent studies), EoS calibrated with other methods such as ultrasonic interferometry, and experimental studies using an LVP, to ensure accuracy in the lower-pressure range up to 20 GPa. While some materials have been repeatedly investigated (*e.g.* MgO, NaCl, Pt…), not all materials have received equal attention in the literature, such as CsCl, KBr, Re and Ni. The comprehensive evaluation of all published equations of state and their different formalisms is beyond the scope of this short communication. However, for any inconsistent results in the pressure calculations, alternative references were explored, such as for molybdenum. The results demonstrate that the most promising *PT* calibrants should include materials with high compressibility and large thermal pressure, such as CsCl and Pt, respectively.

## 2. Methods

### 2.1. Experimental procedure

High-pressure experiments were carried out at the P61B endstation at PETRA III using the Aster-15 LVP. For a description of the beamline station and experimental methods, see Farla *et al.* (2022 ▸).

Briefly, powders of all materials (Table 1 ▸) were purchased with at least 99.9% purity, and pairs of *PT* calibrants were carefully chosen, intimately mixed in a mortar with acetone with mixing ratios based on the reference intensity ratio method, and stored in a vacuum oven. Three experiments were carried out in nearly identical cell assemblies containing three to five samples. The ‘control’ experiment (BT654) included a thermocouple (C-type, W5%Re/W26%Re), and is primarily presented here, whereas results for the other two experiments without thermocouple can be found in the online supporting information (Figs. S1 to S3). The pressure effect on the electromotive force of a C-type thermocouple is small (<15 K up to 10 GPa; Li *et al.*, 2003 ▸), and so thermocouple temperatures are not ‘corrected’ here. In all experiments, four heating cycles at identical steps of DC power were carried out at increasing press loads (1.09 MN, 2.90 MN, 4.60 MN and 6.29 MN). At each target, the parameters of press load and heating power together with corresponding diffraction patterns were collected for each sample in the assembly with acquisition times ranging from 50 to 120 s using the Ge detector at P61B. The duration of spectra acquisition is sample dependent. All diffraction data were processed by fitting each XRD pattern using *PDIndexer* (Seto *et al.*, 2010 ▸) to obtain the lattice parameters/unit-cell volumes of each material along with associated fitting errors (Tables S1 to S3).

### 2.2. Software development

A custom software package, called *EosCross*, was written to enable quick determination of pressure and temperature during beam time by processing obtained lattice parameters and unit-cell volumes, obtained by fitting diffraction patterns. The Python-based software can be started with a selection menu script. The user is then presented with a graphical user interface (GUI) containing radio buttons for each *PT* calibrant pair. For every selection of *PT* calibrants, a Python sub-process is launched for the corresponding script (Base_Mat1+Mat2.py), which opens a new window. This window is then used to input the lattice parameters or unit-cell volume of the material pair for joint calculation of pressure and temperature (Fig. S4). This process is accomplished by calling on two individual scripts, one specific to each material (Mat1.py and Mat2.py). By editing these material scripts, different combinations of materials or equations of state of the same materials can be tested using the same GUI. The software employs the implementation of the Burnman code, developed by Cottaar *et al.* (2016 ▸). For more information, visit the Gitlab DESY website: https://gitlab.desy.de/robert.farla/eoscross.

*EosCross* includes the formalisms (via Burnman) and parameters for many equations of state of materials used in this experimental investigation (Table 1 ▸), as well as additional equations of state for Au (Matsui, 2010 ▸), SiC (Wang *et al.*, 2016 ▸), Ir (Anzellini *et al.*, 2021 ▸), hBN (Godec *et al.*, 2000 ▸) and geo-materials such as olivine, stishovite and corundum (Stixrude & Lithgow-Bertelloni, 2011 ▸). For the materials Ir, Re, Pt, Au, Mo, MgO, KCl and KBr, the Burnman code was modified for *EosCross* by adding entries of formalisms used in the manuscripts of the published EoS without overwriting the standard formalisms provided by Burnman. The pressure calculations for each material were cross-checked between the software and the published results in each paper to ensure the calculations are done correctly. Using the lattice parameters/unit-cell volumes and corresponding uncertainties from peak fitting, *EosCross* calculates pressure at any chosen temperature for a material, as well as the combined pressure and temperature for a pair of materials. This joint *PT* calculation is performed by plotting the isochors of both equations of state in *PT* space and finding the intersection, including the confidence interval shown as lightly shaded intersecting bands (Fig. S4). For best results with small error bars: (i) uncertainties used from peak fitting need to be extremely small (no stress effects – particularly obvious when using angle-dispersive XRD, sharp peaks,…) and (ii) the two isochors should have strongly different slopes. For example, if two materials have equations of state that produce sub-parallel isochors, the errors in the joint *PT* estimation will still be very large, despite very good data quality (Fig. S4).

## 3. Results and interpretations

**Figure 1:**
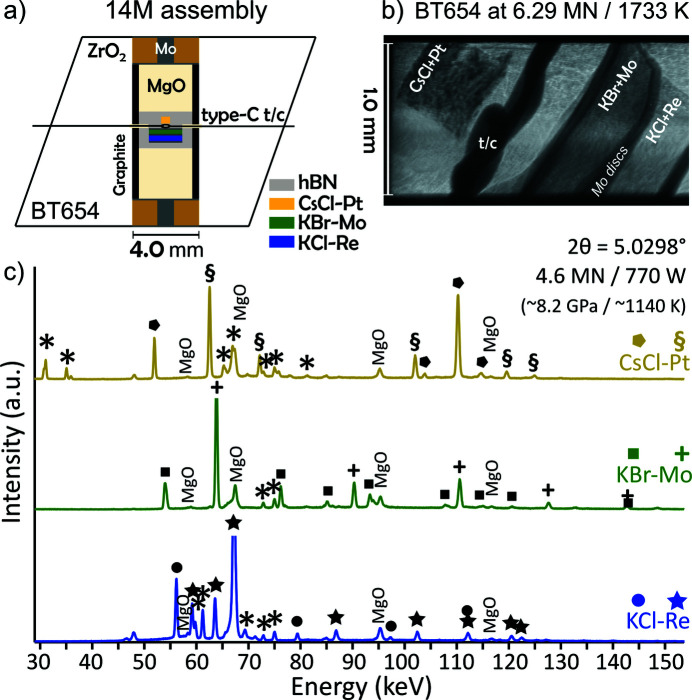
(*a*) Cell assembly containing the three pairs of *PT* calibrants, CsCl–Pt, KCl–Re and KBr–Mo and thermocouple. (*b*) X-ray radiograph showing the samples *in situ* at high *PT* conditions. (*c*) Representative energy-dispersive XRD patterns of selected calibrants at the indicated conditions. Fluorescence lines, including those from the Pb shielding of the detectors, are indicated by asterisks (*). MgO peaks from the pressure medium can be identified.

The cell assembly in this study was imaged by *in situ* X-ray radiography (Fig. 1 ▸) and representative energy-dispersive XRD patterns (of BT654) demonstrate the high data quality produced by the Ge detectors at beamline station P61B (Fig. 1 ▸). Hundreds of acquired diffraction patterns were fitted to extract the lattice parameters/unit-cell volumes used to calculate pressures at the thermocouple temperature for each material in each sample (Tables S1–S3). Obtaining accurate, unbiased, unit-cell volumes is a matter of debate when the availability of distinguishable peaks for fitting varies due to experimental conditions, such as grain growth in the halides (disappearance of peaks), overlap with Pb fluorescence, and peak overlap caused by shifts as a result of pressure and temperature changes. In this study, all unaffected distinguishable peaks in the diffraction data were included in the fitting in order to minimize errors in the unit-cell volume determinations. However, there is some concern that this may lead to a ‘bias’ in the volume determinations, as some peaks were not consistently included in the fitting due to the above-mentioned conditions. To check for this bias, the diffraction data of experiment BT654 are refitted a second time using only the same peaks available in all diffraction patterns (Table S5). We conclude that the presented volume data (Tables S1, S2 and S3) are not biased by including all available peak information, where possible.

**Figure 2:**
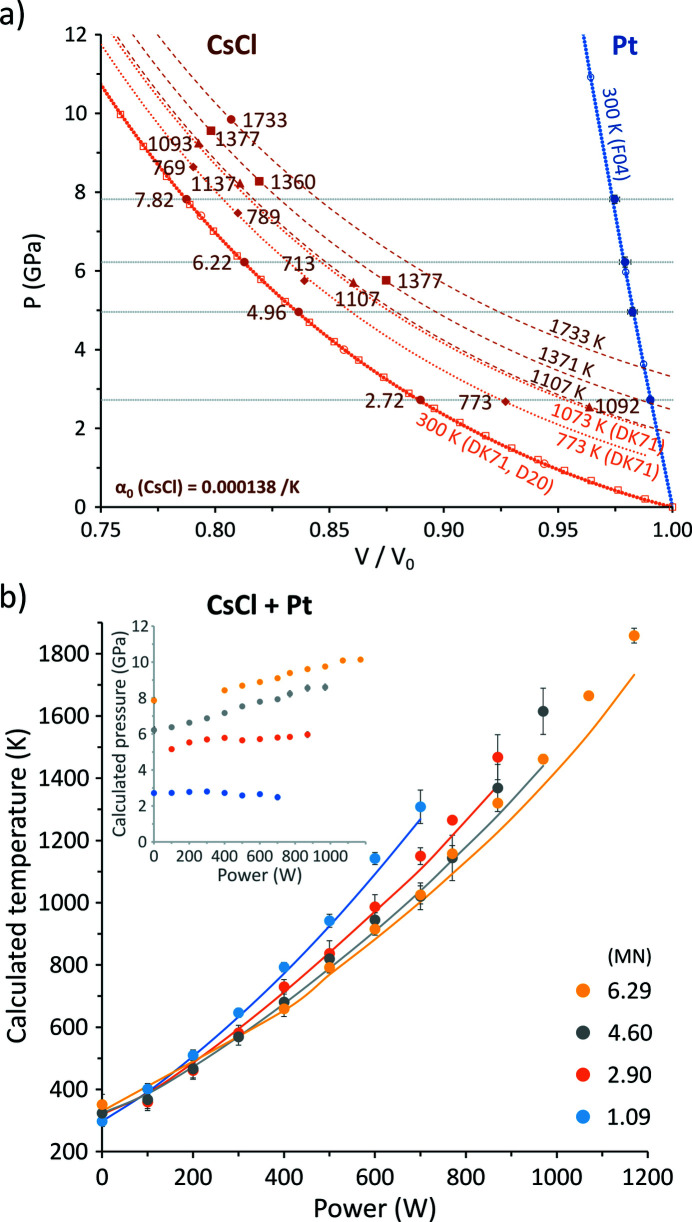
(*a*) *P*–*V*/*V*_0_ diagrams calculated for CsCl and Pt using thermocouple temperatures (see supplementary online materials for more). Various curves (light orange, blue) and data points (open symbols) from previous studies are included and referenced in brackets. DK71: Decker (1971 ▸); D20: Dewaele (2020 ▸); F04: Fei *et al.* (2004 ▸). (*b*) Power–temperature curves (solid lines) from the thermocouple. Data points represent joint calculations of *P* (inset) and *T* using equations of state of each material (see Table 1 ▸) and measured unit-cell volumes at given press load (MN) and heating power (W).

Pressure data are plotted against the normalized unit-cell volume *V*/*V*_0_, where *V*_0_ is the volume of a material under ambient conditions, and expectedly show a striking difference in compressibility between the chosen halide/metal pair [Fig. 2 ▸(*a*), Figs. S5(*a*), S5(*c*) and S5(*e*)]. The steeper the curve, the more incompressible the material. Temperature counteracts compressibility due to volume thermal expansion, as indicated by the dotted and dashed curves for the various halides, although this effect is reduced at higher pressures. One important aspect to note is that, although all three halides CsCl, KCl and KBr are highly compressible and exert low thermal pressures, there are differences up to 0.3 GPa in their calculated pressures at the same thermocouple temperatures [Fig. S6(*a*)]. The differences in calculated pressures among the metals Pt, Re and Mo are expectedly worse [Fig. S6(*b*)]. These variations arise from the uncertainties in the equations of state used, as well as possible stress, pressure and temperature gradients in the assembly, which vary with press load and heating power.

Correlations between pressure and temperature are calculated using *EosCross* for the three sample pairs and are compared with the actual thermocouple power–temperature curves [Fig. 2 ▸(*b*), Figs. S5(*b*), S5(*d*) and S5(*f*) and Table S4]. The extent to which the accuracy of the results relies on the metal phase seems to be significant, as the changes in *d*-spacing for the lattice *hkl* due to thermal pressure are not as substantial as those resulting from compressibility. Notwithstanding, Pt, Mo and Re perform quite well, and the temperatures from the joint *PT* calculations at each press load increment follow the thermocouple curves reasonably well, particularly for CsCl + Pt [Fig. 2 ▸(*b*)].

Following this we see that, at the highest DC power, calculated temperatures deviate more strongly from the measured thermocouple temperatures [*e.g.* Fig. 2 ▸(*b*)] than at moderate heating power. Systematic high-temperature deviations may be due to a poorer constraint on the temperature derivative in the used equations of state, whereas any scatter likely results from the disappearance of peaks as grain growth becomes an issue for the energy-dispersive point detector.

Deviations in pressures among pairs of materials particularly show up in the low-temperature range. The likely reason is that, after each increase in press load, new stresses could have built up again, which required some temperature to relax. For future consideration, additional annealing stages in the experiment would likely offer better, less scattered, pressure data, particularly in the lower-temperature ranges. Other issues may occur when one phase is substantially stronger than the other in a mixture. The strong phase (*e.g.* MgO) may act as the load-bearing framework, with a weak phase (NaCl) mixed in. A consequence, particularly at lower temperatures, is that the pressure is not homogeneous in the aggregate under high press load. The strong phase may report higher than average (true) pressures, whereas the weak phase will report lower than average pressures [see Figs. S7(*a*) and S7(*b*)]. In the halide–metal mixtures, this effect of a load-bearing strong phase generally would not apply as long as the weak phase (the halide) is volumetrically more abundant. Furthermore, the metal and the halide phases will also anneal at different temperatures.

Lastly, we compare the compressibility and thermal pressure of the halides in this study (Fig. S9). We show that KBr and CsCl are the most compressible, followed by KCl and finally NaCl as the least compressible. NaCl also exhibits the highest thermal pressure. For an improved resolution on the pressure, less than 0.1 GPa, one could argue that CsCl and KBr are superior to NaCl. For practical considerations, other factors such as X-ray absorption, the B1 to B2 transition pressure (or lack thereof for CsCl), melting curve, grain growth kinetics and peak positions help deciding what material to use as an internal *P* (*T*) calibrant.

### 3.1. Error analysis

In order to obtain a better overview of the performance of the tested *PT* calibrants, we carried out an error assessment for BT654, which included a thermocouple. Note that the equations of state we used inherently also contain uncertainties in the parameters. However, due to missing information in several publications (Tateno *et al.*, 2019 ▸; Köhler *et al.*, 1997 ▸; Matsui *et al.*, 2009 ▸; Sokolova *et al.*, 2013 ▸) and the challenge of managing the many possible variations in the EoS parameters, errors in the published equations of state were not implemented in the software at this time. However, we briefly explored the impact of a ±1% error in each MGD parameter of CsCl and Pt on the joint *P*,*T* estimations [Fig. S10(*a*)]. The result of this analysis, when only one parameter is changed at a time for both materials with −1% and +1% differences, shows that a large impact comes from *K*_0_, then the Grüneisen parameter γ_0_, followed by the *K*′ parameter. Changing the *q*_0_ and Debye parameters has minimal impact on the joint *P*,*T* calculations. This example shows that a serious increase in *V* errors than measured is not required in order to cover the 1% variations in the EoS parameters (Tables S1, S2, S3 and S5). If we consider the extreme ±1% errors in the *K*_0_, *K*′ and γ_0_ parameters combined, then clearly larger deviations from the ‘true’ pressure and temperature will be estimated [Fig. S10(*a*)]. Notwithstanding, by far the largest effect on the joint *P*,*T* calibration comes from inaccurate *V*_0_ values, particularly for the metal (*i.e.* Pt), and thus wildly different joint *P*,*T* estimations can be obtained [Fig. S10(*b*)].

**Figure 3:**
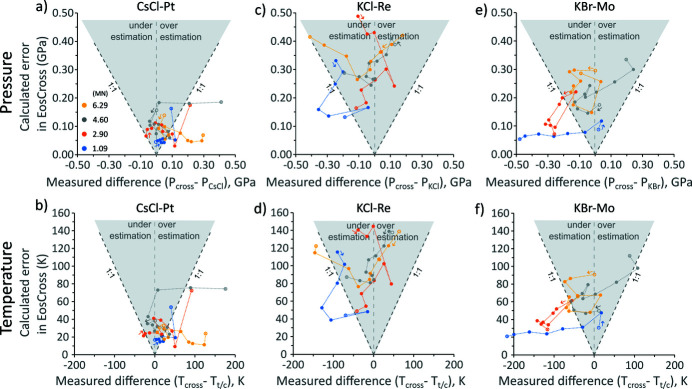
(*a*–*b*, *c*–*d*, *e*–*f*) Evaluation of *P* and *T* differences for each pair of calibrants in experiment BT654. The *y*-axis shows the errors from the joint *P* and *T* calculation using the custom software (*EosCross*; see online supporting information). The *x*-axis shows the difference between the *P* and *T* calculated using the software and the *P* and *T* constrained by the thermocouple temperature. The 1:1 lines indicate a one-to-one agreement. For each colour-coded press load step, the points are connected by dotted lines following the incremental increase in heating power. There is no clear suggestion that peak fitting errors or differences from the thermocouple measurement follow a systematic trend with increasing *P* and *T*. Generally, the more points inside the shaded region and the smaller the differences, the better the choice of *PT* calibrants.

We present a comparison between (i) the calculated differences of the predicted *P* and *T* (using EosCross) and the measured values based on the thermocouple temperature, and (ii) the *P*,*T* errors calculated in *EosCross*, propagated from the measurement errors in the unit-cell volumes of the materials obtained by peak fitting (Fig. 3 ▸). The results show that when the isochors of two materials, *e.g.* CsCl and Pt, have strongly different slopes (Fig. S4) the *PT* estimation is particularly robust and the errors of the joint *PT* estimation can be considerably small in pressure and temperature (Fig. 3 ▸). However, not all data are of equal quality, so there are some deviations [Figs. 3 ▸(*a*) and 3(*b*)]. Using the data shown on the *x*-axes in Fig. 3 ▸, also tabulated in Table S4, the averages of the absolute differences between |*P*_halide_ − *P*_cross_| and |*T*_t/c_ – *T*_cross_| are calculated, following the same strategy employed by Crichton & Mezouar (2002 ▸). For the CsCl–Pt pair, the average deviation in *P* and *T* are obtained as 0.087 GPa and 37 K (Table S4). Given the unavoidable presence of temperature and pressure gradients and possible stress contributions in the cell assembly, these results for the CsCl–Pt pair are encouraging.

The KCl–Re pair appears to perform equally well with overall acceptable deviations from the thermocouple temperatures and derived pressures [Figs. 3 ▸(*c*) and 3(*d*)]. *EosCross* predicts larger errors in the joint *P*,*T* calculation for this pair, because rhenium has a relatively small unit-cell volume (*V*_0_ = 29.428 Å^3^) in comparison with the other materials, so a better resolution is needed (Tables S2 and S3). Rhenium is also comparatively stiff and is the only phase used in this study with a hexagonal close-packed structure, which means its volume cannot be determined from a single peak. For the KCl–Re pair, the average deviations in *P* and *T* are 0.118 GPa and 43 K (Table S4), respectively.

The last pair, KBr–Mo, appears to give the poorest results with both larger errors from *EosCross* and larger deviations when the jointly calculated temperatures and pressures are compared with the thermocouple temperatures and derived pressures [Figs. 3 ▸(*e*) and 3(*f*)]. At 1.09 MN press load, calculated KBr pressures are lower towards higher thermocouple temperatures than those of CsCl and KCl [Fig. S6(*a*)], which may suggest it was not stable in the high-pressure B2 structure [the B1–B2 transition occurs around 2.3 GPa at room temperature (Dewaele *et al.*, 2012 ▸)]. Furthermore, there is some concern about the many equations of state published for Mo, tabulated by Huang *et al.* (2016 ▸). While pressures calculated at 300 K are in reasonable agreement, pressures calculated at high temperatures disagree by up to 1 GPa for Mo [*e.g.* Fig. 5 of Huang *et al.* (2016 ▸)]. Here, the best results were obtained using parameters published by Sokolova *et al.* (2013 ▸) (Table 1 ▸), and calculated pressures are in reasonable agreement with those obtained from the halides at 4.60 MN and 6.29 MN over the whole temperature range, but show a stronger systematic deviation of over 0.3 GPa [Fig. S6(*b*)] at 1.09 MN and 2.90 MN. For the KBr–Mo pair, the average deviations in *P* and *T* are 0.157 GPa and 71 K (Table S4), respectively.

If the uncertainties surrounding molybdenum can be resolved, it can be a promising candidate as a *PT* calibrant due to its lower atomic number. It is less X-ray absorbing, and for energy-dispersive X-ray diffraction its characteristic X-rays (*i.e.* fluorescence lines) are conveniently at low energies and do not interfere with diffraction peaks of any material. It exhibits a decent thermal pressure. Nickel is another promising candidate, because it exhibits a strong thermal pressure, possibly second to platinum as shown by the slopes of the isochors in *EosCross*. However, joint *PT* estimations do not appear to be very reliable using current published thermal equations of state of nickel [Fig. S5(*f*)]. Our data unambiguously show that, in order to obtain the most precise simultaneous *PT* evaluation from diffraction data, the materials presented in this study should be improved, revisited and remeasured with greater precision.

## 4. Conclusions

In this study, a large range of materials were explored as paired mixtures to test their feasibility for use as promising *PT* calibrants. For a successful application to jointly estimate pressure and temperature *in situ* using X-rays, each material should be stable and unreactive over large *PT* ranges, should have a simple (cubic) crystal system, produce strong reflections, and pairs should exhibit a strong contrast in high compressibility (small *K* and **K*′* values for good pressure resolution) and in thermal pressure (very different α*K*_*T*_ values). With the aid of the custom-written software *EosCross*, rapid feedback on the pressure and temperature in an HPHT cell can be obtained from a joint calculation during the experiment. The most promising combinations of materials CsCl–Pt, KCl–Re and KBr–Mo were tested in an assembly with a thermocouple, which gave good agreements between the cross-calibrated *P* and *T* values and those of a thermocouple and pressures calculated based on the thermocouple temperatures. For all four heating runs (BT654), the average deviations in *T* calculated for the three pairs are 37 K (CsCl–Pt), 43 K (KCl–Re) and 71 K (KBr–Mo). The average deviations in *P* calculated for the three pairs are 0.087 GPa (CsCl–Pt), 0.118 GPa (KCl–Re) and 0.157 GPa (KBr–Mo). These results are consistent with the errors calculated by *EosCross*, following the trend where the smallest errors/deviations in *P* and *T* are expected for isochors intersecting at the largest angle (*i.e.* CsCl–Pt as the best estimation) and larger errors/deviations in *P* and *T* where the isochors intersect at smaller angles (*i.e.* KBr–Mo as the worst estimation of the three α*K*_*T*_ pairs). Other combinations of materials explored in experiments under nearly identical conditions (same cell assembly, same heating and load steps) without a thermocouple produced similar consistent trends in pressures and temperatures. These results point towards the conclusion that, today, joint pressure and temperature estimations by *in situ* XRD can provide satisfactory results with good confidence for HPHT experiments and offer a viable alternative to an invasive thermocouple in the assembly.

## 5. Data availability

The data that support the findings of this study are available within the article and its supporting information. The software *EosCross* is freely available from the DESY GitLab: https://gitlab.desy.de/robert.farla/eoscross.

## 6. Related literature

The following references, not cited in the main body of the paper, have been cited in the supporting information: Hirao *et al.* (2022 ▸); Litasov *et al.* (2013*b* ▸); Walker *et al.* (2002 ▸).

## Supplementary Material

Supplementary Figures S1 to S10, Tables S1 to S5. DOI: 10.1107/S1600577523004538/vl5008sup1.pdf

## Figures and Tables

**Table 1:** Equation of state parameters of materials used to jointly calculate pressure and temperature *K*_0_, 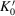 and 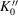 are the bulk modulus and pressure derivatives under ambient conditions. Θ_0_ is the Debye temperature. The volume dependence of the Grüneisen constant is expressed as γ = γ_0_(*V*/*V*_0_)^*q*^. Temperatures and pressures given in quotation marks indicate maximum *P* and *T* ranges from a calculation in these studies (*i.e.* not experimental *P* and *T*).

Material	Reference	*K*_0_ (GPa)	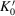	Θ_0_ (K)	γ_0_	*q*	α*K*_*T*_ (GPa K^−1^)	Model used	Technique	Maximum *P* *T*
NaCl	Matsui *et al.* (2012 ▸)	23.7	5.14†	279	1.56	0.96	–	MGD	UI + ED-XRD in LVP	12 GPa, 673 K
KCl	Tateno *et al.* (2019 ▸)	17.4	5.77	–	1.8	0.7	0.0033	MGD	AD-XRD in LH-DAC	61 GPa, 2600 K
KBr	Köhler *et al.* (1997 ▸)	17	5.38	–	–	–	0.0022	Vinet + thermal	ED-XRD in DAC	45 GPa, 300 K
CsCl	Decker (1971 ▸)	17‡	5.4§	151	1.99	1.18	–	MGD	Lattice vibration calculations	‘43 GPa’, ‘1073 K’
MgO	Tange *et al.* (2009 ▸)¶	160.63	4.367	761	1.442	1.1	–	MGD/Vinet	Unified analyses	‘196 GPa’, ‘3700 K’
Pt	Matsui *et al.* (2009 ▸)	273.9	5.2	230	2.7	1.1	–	MGD	ED-XRD in LVP	42 GPa, 1600 K
Ni	Campbell *et al.* (2009 ▸)	179	4.3	415	2.5	1	–	MGD	ED-XRD in LVP + AD-XRD in DAC	65 GPa, 2500 K
Mo	Sokolova *et al.* (2013 ▸)	249	4.47	470	1.98	1.99	–	MGD	Unified analyses	‘300 GPa’, ‘3500 K′
W	Litasov *et al.* (2013*a* ▸)	317	3.16	370	1.85	1.08	–	MGD	ED-XRD in LVP	33.5 GPa, 1673 K
Re	Zha *et al.* (2004 ▸)	360	4.5	–	–	–	0.00776††	BM3 + thermal	ED-XRD in IRH-DAC	8.5 GPa, 1900 K

† Including 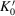 (GPa^−1^) = −0.392.

‡ Updated from Köhler *et al.* (1997 ▸).

§ Not reported by Decker (1971 ▸). Obtained by fitting recent room-temperature data by Dewaele (2020).

¶ Including parameters *a* = 0.138 and *b* = 5.4 in the expression of the volume dependence of γ.

†† Including (∂*K*_*T*_/∂*T*)_*V*_ = −0.00815 GPa K^−1^.
