# Supplementary material for: Towards joint in situ determination of pressure and temperature in the large volume press exclusively from X-ray diffraction
Source: J Synchrotron Radiat. 2023 Jun 21;30(Pt 4):807–14. doi: 10.1107/S1600577523004538 (PMC10325023; doi:10.1107/S1600577523004538)
Supplement: Supplementary file 1 [file s-30-00807-sup1.pdf]

JOURNAL OF
SYNCHROTRON
RADIATION

Volume 30 (2023)

Supporting information for article:

Towards joint *in situ* determination of pressure and temperature in the large volume press exclusively from X-ray diffraction

Robert Farla

Table S1. Experimental conditions and unit cell volumes obtained by *in situ* X-ray diffraction for **BT600**. Volumes are in Å^3, pressures in GPa. 2θ = 7.2243(31)°

Load (MN)	P (W)	T (K, est.)	V_{NaCl}	P_{NaCl}	V_{Ni}	P_{Ni}	V_{NaCl}	P_{NaCl}	V_{MgO}	P_{MgO}	$V_{KCl (B2)}$	$P_{KCl (B2)}$	V_{Pt}	P_{Pt}	V_{NaCl}	P_{NaCl}	V_{Mo}	P_{Mo}
BenchXRD*:	-	300	179.4252	0	43.7727	0	179.4252	0	74.6823	0	54.5†	0	60.3793	0	179.4252	0	31.1723	0
ED- XRD:	-	300	179.46(6)	0	43.77(4)	0	179.54(12)	0	74.70(2)	0	n/a	0	60.38(2)	0	179.4(1)	0	31.16(1)	0
1.09	0	300	163.31(20)	2.83	43.164(7)	2.585	162.34(6)	3.052	73.59(6)	2.448	48.63(3)	2.76	59.81(2)	2.659	163(14)	2.899	30.851(6)	2.634
	100	400	164.43(13)	2.85	43.349(12)	2.616	163.43(5)	3.07	73.87(9)	2.291	48.95(2)	2.81	59.94(3)	2.749	163.73(9)	3.002	30.893(7)	2.743
	200	510	165.56(8)	2.93	43.534(19)	2.879	165.03(4)	3.045	73.99(6)	2.657	49.27(2)	2.93	60.11(2)	2.834	165.21(7)	3.007	30.897(8)	2.876
	300	640	167.7(19)	2.86	43.742(18)	3.184	167.26(5)	2.945	74.26(3)	2.789	50.41(1)	2.63	60.32(2)	2.843	167.77(7)	2.844	31.03(13)	2.826
	400	780	170.09(10)	2.81	44.007(9)	3.423	169.67(4)	2.885	74.82(2)	2.425	50.77(9)	2.83	60.56(2)	2.869	169.85(2)	2.853	31.095(4)	3.019
	500	930	172.03(17)	2.91	44.429(34)	3.229	172.43(15)	2.84	75.13(1)	2.718	51.4(14)	2.94	60.82(2)	2.922	172.49(13)	2.831	31.208(10)	2.916
	600	1100	175.46(6)	2.83	44.819(25)	3.347	175.27(11)	2.861	75.61(2)	2.757	52.48	2.92	61.12(4)	2.942	174.91(24)	2.917	31.253(8)	3.068
	700	1270	177.21(10)	3.08	45.374(55)	3.068	178.6(21)	2.89	76.09(1)	2.915	-	-	-	-	178.07(15)	2.962	31.36(5)	3.544
2.90	0	320	154.02(5)	5.35	42.732(12)	4.707	155.03(20)	5.049	72.35(2)	5.547	45.49(4)	5.34	59.17(1)	5.975	153.49(8)	5.52	30.535(9)	5.48
	100	390	153.55(6)	5.70	42.778(13)	5.12	154.6(12)	5.377	72.39(2)	5.813	45.4(4)	5.63	59.22(1)	6.264	152.81(21)	5.938	30.524(8)	5.914
	200	490	153.2(7)	6.10	42.861(19)	5.63	154.03(7)	5.836	72.51(3)	6.053	45.29(4)	6.01	59.32(1)	6.531	152.41(17)	6.351	30.534(7)	6.304
	300	600	153.17(8)	6.42	42.984(20)	6.07	154(10)	6.161	72.62(2)	6.413	45.4(3)	6.21	59.48(2)	6.564	152.81(18)	6.535	30.58(7)	6.43
	400	720	153.7(6)	6.60	43.182(19)	6.293	154.59(7)	6.324	72.89(3)	6.467	45.61(3)	6.34	59.65(3)	6.646	153.68(8)	6.603	30.635(6)	6.551
	500	840	154.62(8)	6.68	43.475(3)	6.212	155.12(12)	6.533	73.18(2)	6.525	45.91(4)	6.41	59.87(4)	6.565	154.63(4)	6.681	30.696(5)	6.663
	600	980	155.75(8)	6.73	43.728(7)	6.394	156.03(12)	6.647	73.51(3)	6.578	46.25(6)	6.48	60.06(3)	6.686	155.74(6)	6.732	30.763(7)	6.763
	700	1110	157.06(5)	6.75	44.026(18)	6.472	157.45(3)	6.638	73.88(2)	6.569	46.69(4)	6.48	60.3(4)	6.649	157.18(7)	6.714	30.826(6)	6.935
	770	1220	158.37(7)	6.71	44.271(13)	6.544	158.75(3)	6.606	74.18(2)	6.574	47.11(7)	6.45	60.5(4)	6.608	158.16(17)	6.764	30.879(10)	7.065
4.60	0	320	148.88(6)	7.08	42.458(6)	6.03	150.01(8)	6.692	71.68(3)	7.327	43.8(3)	7.18	58.89(3)	7.465	148.76(5)	7.142	30.356(5)	7.117
	100	390	148.66(4)	7.36	42.523(2)	6.292	149.61(2)	7.017	71.68(1)	7.663	43.76(3)	7.41	58.89(3)	7.986	148.52(4)	7.414	30.359(9)	7.392
	200	480	148.43(6)	7.70	42.677(3)	6.357	149.56(9)	7.288	71.79(2)	7.83	43.7(4)	7.73	59.03(4)	7.912	148.34(9)	7.733	30.372(8)	7.704
	300	570	148.12(4)	8.10	42.775(2)	6.792	148.98(4)	7.78	71.87(2)	8.194	43.61(2)	8.12	59.03(3)	8.617	147.86(6)	8.197	30.374(8)	8.165
	400	680	147.77(6)	8.54	42.858(3)	7.392	148.47(7)	8.28	71.93(2)	8.677	43.53(2)	8.52	59.12(3)	8.979	147.29(5)	8.727	30.363(8)	8.792
	500	790	147.39(5)	9.03	42.961(5)	7.985	147.99(4)	8.796	72.01(1)	9.144	43.44(2)	8.97	59.2(3)	9.483	147.1(9)	9.138	30.368(5)	9.32
	600	910	147.35(5)	9.40	43.098(5)	8.497	147.71(3)	9.256	72.22(2)	9.343	43.49(2)	9.25	59.33(3)	9.717	147.21(7)	9.452	30.393(5)	9.692
	700	1040	148.04(8)	9.52	43.314(9)	8.766	148.25(3)	9.437	72.48(2)	9.505	43.72(2)	9.34	59.5(4)	9.896	147.87(8)	9.578	30.462(7)	9.737
	770	1140	148.72(7)	9.55	43.497(15)	8.914	149.16(4)	9.386	72.72(2)	9.518	43.96(2)	9.33	59.67(4)	9.864	148.78(9)	9.527	30.512(11)	9.796
	870	1290	150.07(6)	9.49	43.784(6)	9.096	150.33(4)	9.396	73.12(2)	9.453	44.44(2)	9.20	59.93(4)	9.731	150.27(3)	9.42	30.591(4)	9.844
6.29	0	330	144.17(8)	8.98	42.125(9)	7.715	144.75(4)	8.736	70.98(2)	9.28	42.4(2)	9.05	58.56(2)	9.075	144(8)	9.056	30.159(4)	8.994
	400	660	144.06(8)	9.99	42.608(16)	8.369	144.77(5)	9.69	71.33(2)	10.15	42.41(2)	9.98	58.85(3)	10.25	143.89(8)	10.066	30.189(8)	10.313
	500	770	144.03(6)	10.34	42.733(13)	8.831	144.77(4)	10.023	71.51(2)	10.345	42.51(2)	10.17	58.97(3)	10.5	144.09(11)	10.313	30.216(6)	10.621
	600	890	144.12(5)	10.64	42.855(12)	9.331	144.72(4)	10.382	71.66(1)	10.643	42.55(3)	10.43	59.1(3)	10.725	144.2(8)	10.603	30.249(5)	10.884
	700	1010	144.39(5)	10.88	43.027(20)	9.685	144.77(5)	10.718	71.9(2)	10.769	42.62(2)	10.68	59.21(3)	11.084	144.51(7)	10.831	30.286(7)	11.15
	770	1100	144.72(6)	11.01	43.178(12)	9.868	145.11(4)	10.848	72.04(1)	10.977	42.72(2)	10.81	59.27(2)	11.475	144.75(7)	10.996	30.32(7)	11.298
	870	1230	145.37(4)	11.14	43.393(2)	10.212	145.74(3)	10.987	72.32(2)	11.111	42.9(2)	10.96	59.44(2)	11.681	145.23(5)	11.2	30.366(7)	11.565
	970	1380	146.28(11)	11.22	43.666(4)	10.495	146.42(4)	11.165	72.59(2)	11.372	43.1(1)	11.13	59.69(2)	11.69	145.89(6)	11.38	30.426(5)	11.801
	1070	1550	147.36(15)	11.29	44.057(5)	10.501	147.49(4)	11.239	72.99(1)	11.449	43.48(4)	11.11	59.98(2)	11.634	147.42(7)	11.265	30.53(5)	11.74
	1170	1740	149.18(5)	11.16	44.581(4)	10.28	149.57(7)	11.014	73.61(1)	11.161	44.37(5)	10.58	60.48(2)	10.692	150.33(5)	10.742	30.703(8)	11.221

* The unit cell volumes at room pressure and temperature conditions were obtained using Match! Software with FullProf from diffraction data obtained in the GNR Europe θ-θ benchtop diffractometer at P61B.
† Extrapolated V_0 for B2 structure (Dewaele *et al.* 2012)
The following peaks were routinely included in the fit using PDIndexer:
NaCl: 200,220,222,420,422
Ni: 111,200,220
MgO: 111,200,220,311,222,400,420
KCl (B2): 110,200,211,220,321
Pt: 111,200,220,311,222,400,420,422,511
Mo: 110,200,211,220,310,321

Table S2. Experimental conditions and unit cell volumes obtained by in situ X-ray diffraction for **BT623**. Volumes are in Å^3, pressures in GPa. 2θ = 5.0236(6)°

Load (MN)	P (W)	T (K, est.)	V_{CsCl}	P_{CsCl}	V_W	P_W	V_{NaCl}	P_{NaCl}	V_{Pt}	P_{Pt}	$V_{KBr\ (B2)}$	$P_{KBr\ (B2)}$	V_{Re}	P_{Re}
BenchXRD*:	-	300	70.0874	0	31.7117	0	179.4252	0	60.3793	0	62.93†	0	29.4280	0
ED-XRD:	-	300	70.42(5)	0	31.73(3)	0	179.58(8)	0	60.38(5)	0	n/a	0	29.41(5)	0
1.09	0	300	61.89(4)	2.96	31.42(7)	2.976	162.85(20)	2.935	59.89(2)	2.266	55.51(2)	2.98	29.19(4)	3.039
	100	396	62.276(4)	2.98	31.462(8)	2.97	164.29(16)	2.878	60.1(2)	2.239	55.91(2)	2.97	29.26(5)	2.874
	200	511	62.94(6)	2.93	31.533(7)	2.787	165.82(10)	2.876	60.29(2)	2.223	56.6(1)	2.86	29.33(4)	2.861
	300	638	63.87(2)	2.81	31.607(5)	2.625	168.32(14)	2.737	60.49(1)	2.317	57.85(4)	2.54	29.43(6)	2.635
	400	778	64.69(1)	2.80	31.673(6)	2.636	170.58(34)	2.72	60.72(3)	2.371	58.57(3)	2.54	29.5(3)	2.841
	500	931	65.8(9)	2.74	31.756(6)	2.542	171.62(28)	2.978	61(2)	2.366	59.32(2)	2.58	29.59(5)	2.949
	600	1097	67.09(14)	2.69	31.834(8)	2.562	-	-	-	-	60.56(5)	2.49	29.7(4)	2.995
	700	1274	68.13(15)	2.79	31.928(4)	2.503	-	-	-	-	61.93(2)	2.448	29.83(5)	2.908
2.90	0	320	57.8(13)	5.56	31.164(9)	5.766	153.77(21)	5.432	59.35(1)	5.293	51.71(4)	5.63	28.99(4)	5.708
	100	391	57.76(12)	5.76	31.181(9)	5.911	153.55(14)	5.704	59.42(1)	5.501	51.6(3)	5.88	29.02(4)	5.889
	200	490	57.58(10)	6.12	31.207(10)	6.093	153.47(14)	6.01	59.5(1)	5.855	51.57(3)	6.13	29.08(4)	5.855
	300	599	57.66(5)	6.31	31.251(7)	6.138	154.04(16)	6.145	59.61(4)	6.147	51.71(4)	6.25	29.15(3)	5.751
	400	717	57.97(5)	6.35	31.292(6)	6.26	154.91(19)	6.227	59.87(1)	5.787	51.79(1)	6.45	29.21(2)	5.979
	500	844	58.35(4)	6.37	31.355(5)	6.207	155.97(29)	6.282	60.12(4)	5.583	52.23(1)	6.37	29.28(2)	6.089
	600	976	58.79(3)	6.37	31.43(8)	6.058	157.38(28)	6.264	60.32(2)	5.681	52.73(2)	6.29	29.36(2)	6.009
	700	1111	59.35(2)	6.31	31.499(6)	5.996	159.27(18)	6.149	60.62(1)	5.413	53.33(2)	6.15	29.46(2)	5.969
	770	1221	59.94(4)	6.19	31.564(7)	5.862	160.51(37)	6.148	60.85(1)	5.265	53.96(1)	5.97	29.53(2)	5.883
4.60	0	324	56.13(4)	6.94	31.058(7)	6.927	149.36(21)	6.921	59.08(3)	6.753	50.47(2)	6.73	28.9(4)	6.992
	100	389	56.08(4)	7.13	31.075(10)	7.039	149.3(12)	7.129	59.12(3)	6.995	50.4(2)	6.94	28.92(4)	7.253
	200	477	55.97(4)	7.43	31.077(8)	7.43	149.26(30)	7.394	59.1787(5)	7.374	50.31(2)	7.22	28.94(4)	7.579
	300	574	55.82(4)	7.79	31.099(11)	7.646	149.09(21)	7.739	59.26(1)	7.697	50.18(2)	7.57	28.96(5)	8.121
	400	680	55.65(5)	8.19	31.097(6)	8.173	148.91(13)	8.116	59.34(2)	8.109	50.05(2)	7.93	29(3)	8.412
	500	794	55.51(3)	8.59	31.113(8)	8.544	148.97(4)	8.429	59.44(3)	8.475	49.99(3)	8.24	29.04(3)	8.641
	600	914	55.54(3)	8.84	31.145(5)	8.771	149.19(3)	8.702	59.58(1)	8.732	50.07(3)	8.42	29.1(3)	8.799
	700	1044	55.79(3)	8.92	31.208(7)	8.71	149.93(2)	8.817	59.81(3)	8.632	50.33(2)	8.46	29.19(3)	8.759
	770	1142	55.98(4)	8.98	31.238(7)	8.869	150.77(12)	8.812	59.99(6)	8.532	50.57(3)	8.45	29.24(3)	8.86
	870	1287	56.52(4)	8.83	31.316(6)	8.744	152.49(7)	8.661	60.26(4)	8.441	51.16(2)	8.25	29.36(3)	8.531
6.29	0	330	54.53(5)	8.48	30.946(9)	8.188	145.08(24)	8.597	58.75(1)	8.537	48.79(2)	8.45	28.82(4)	8.086
	400	661	54.43(3)	9.35	30.999(7)	9.145	146.05(62)	9.163	59.06(2)	9.416	48.78(2)	9.21	28.93(4)	9.177
	500	774	54.4(3)	9.65	31.005(8)	9.613	146.17(54)	9.445	59.14(2)	9.874	48.76(2)	9.47	28.96(3)	9.592
	600	887	54.31(2)	10.00	31.026(9)	9.936	146.14(48)	9.794	59.274(1)	10.055	48.8(4)	9.69	29(3)	9.935
	700	1008	54.34(3)	10.25	31.053(7)	10.212	146.4(30)	10.047	59.44(2)	10.166	48.86(3)	9.89	29.05(4)	10.171
	770	1098	54.42(3)	10.38	31.071(7)	10.445	147.26(26)	9.978	59.58(10)	10.161	48.93(9)	10.00	29.11(4)	10.122
	870	1233	54.62(1)	10.50	31.133(11)	10.427	147.4(16)	10.325	59.77(4)	10.337	49.17(2)	10.05	29.18(3)	10.228
	970	1385	54.79(2)	10.68	31.187(8)	10.581	147.88(39)	10.59	59.926(1)	10.768	-	-	-	-

* The unit cell volumes at room pressure and temperature conditions were obtained using Match! Software with FullProf from diffraction data obtained in the GNR Europe θ-θ benchtop diffractometer at P61B.

† Extrapolated V_0 for B2 structure (Köhler *et al.* 1997)

The following peaks were routinely included in the fit using PDIndexer:

CsCl: 210,220,310,321

W: 110,211,220

NaCl: 111,200,222

Pt: 111,200

KBr (B2): 110,200,220

Re: 002,011,012,110,013,112,021

Table S3. Experimental conditions and unit cell volumes obtained by *in situ* X-ray diffraction for **BT654.** Volumes are in Å^3, pressures in GPa. 2θ = 5.0298(6)°

Load (MN)	P (W)	T (K)	V_{CsCl}	P_{CsCl}	V_{Pt}	P_{Pt}	T (K)	$V_{KBr (B2)}$	$P_{KBr (B2)}$	V_{Mo}	P_{Mo}	T (K)	$V_{KCl (B2)}$	P_{KCl}	V_{Re}	P_{Re}
BenchXRD*:	-	300	70.0874	0	60.3793	0	300	62.93†	0	31.1723	0	300	54.5††	0	29.4280	0
ED-XRD:	-	300	70.11(2)	0	60.45(2)$	0	300	n/a	0	31.17(1)	0	300	n/a	0	29.45(4)	0
1.09	0	300	62.36(1)	2.72	59.795(15)	2.73	300	55.85(3)	2.79	30.837(6)	2.76	300	48.68(3)	2.72	29.19(5)	3.00
	100	392	62.832(25)	2.71	59.955(30)	2.66	396	56.46(2)	2.67	30.907(14)	2.62	399	49.08(4)	2.74	29.23(4)	3.19
	200	507	63.26(2)	2.78	60.121(34)	2.76	511	57.03(2)	2.65	30.964(10)	2.73	516	49.6(2)	2.74	29.31(4)	3.10
	300	634	63.93(2)	2.78	60.344(16)	2.71	638	57.88(2)	2.53	31.038(9)	2.75	642	50.36(5)	2.66	29.39(3)	3.11
	400	773	64.97(2)	2.68	60.617(16)	2.56	778	59(2)	2.36	31.134(8)	2.71	784	51.38(3)	2.54	29.47(2)	3.19
	500	925	66.31(2)	2.55	60.916(24)	2.46	932	60.19(4)	2.26	31.231(8)	2.74	938	52.54(7)	2.45	29.59(1)	2.99
	600	1092	67.54(2)	2.54	61.273(21)	2.26	1097	61.55(4)	2.17	31.339(8)	2.78	1103	53.7(9)	2.46	29.74(2)	2.53
	700	1269	69.59(15)	2.39	61.64(62)	2.17	1274	64.31(3)	1.82	31.503(8)	2.48	1280	56.19(5)	2.15	29.87(2)	2.51
2.90	0	320	58.63(4)	4.96	59.323(24)	5.21	320	52.45(3)	5.04	30.554(13)	5.31	320	46.27(16)	4.61	29.04(5)	5.12
	100	388	58.47(2)	5.23	59.392(22)	5.38	391	52.33(3)	5.29	30.558(9)	5.61	395	46.12(12)	4.96	29.08(4)	5.33
	200	486	58.28(5)	5.59	59.438(21)	5.89	490	52.34(3)	5.51	30.582(8)	5.88	495	46.03(6)	5.32	29.13(4)	5.40
	300	595	58.43(5)	5.73	59.624(22)	5.81	599	52.54(3)	5.60	30.636(9)	5.95	603	46.23(3)	5.44	29.18(5)	5.44
	400	713	58.8(4)	5.75	59.843(22)	5.66	718	52.89(3)	5.59	30.708(9)	5.92	722	46.56(2)	5.49	29.27(3)	5.56
	500	840	59.37(3)	5.66	60.047(45)	5.68	844	53.14(12)	5.69	30.791(11)	5.85	849	46.96(2)	5.51	29.34(4)	5.53
	600	972	59.82(2)	5.69	60.287(44)	5.61	976	53.59(12)	5.67	30.861(8)	5.95	979	47.22(4)	5.67	29.4(3)	5.66
	700	1107	60.31(2)	5.70	60.583(31)	5.34	1112	54.09(11)	5.64	30.946(8)	5.94	1116	47.65(5)	5.72	29.47(3)	5.88
	770	1216	60.7(2)	5.73	60.753(8)	5.45	1221	54.55(7)	5.59	31.012(7)	5.98	1226	47.86(4)	5.87	29.53(2)	5.97
	870	1377	61.31(1)	5.76	61.099(93)	5.25	1384	55.5(1)	5.39	31.149(10)	5.74	1392	48.76(4)	5.71	29.65(2)	5.93
4.60	0	324	56.97(9)	6.22	59.13(29)	6.22	324	50.68(10)	6.54	30.443(21)	6.32	324	44.83(1)	6.02	28.99(5)	5.79
	100	386	56.89(5)	6.43	59.162(32)	6.52	389	50.69(9)	6.67	30.47(20)	6.38	392	44.83(2)	6.20	29.01(5)	6.02
	200	473	56.87(4)	6.65	59.261(32)	6.68	477	50.75(8)	6.82	30.486(16)	6.66	481	44.84(1)	6.44	29.04(4)	6.32
	300	570	56.86(2)	6.88	59.367(29)	6.89	574	50.73(7)	7.05	30.498(13)	7.04	579	44.83(1)	6.74	29.07(4)	6.71
	400	675	56.82(4)	7.16	59.481(31)	7.13	680	50.72(7)	7.29	30.521(13)	7.37	685	44.82(2)	7.05	29.1(4)	7.11
	500	789	56.77(1)	7.47	59.626(35)	7.30	794	50.69(7)	7.57	30.558(18)	7.62	799	44.82(1)	7.38	29.14(3)	7.47
	600	909	56.81(1)	7.71	59.773(27)	7.52	914	50.71(6)	7.82	30.594(16)	7.91	920	44.83(1)	7.70	29.19(3)	7.72
	700	1038	56.85(0)	7.97	59.869(51)	8.07	1044	50.78(6)	8.04	30.635(14)	8.21	1050	44.89(2)	8.01	29.25(3)	8.01
	770	1137	56.83(2)	8.22	60.01(85)	8.18	1142	50.86(5)	8.19	30.679(12)	8.34	1147	45.04(1)	8.14	29.3(3)	8.18
	870	1281	57.08(1)	8.35	60.326(52)	7.86	1288	51.21(5)	8.20	30.776(12)	8.27	1294	45.39(4)	8.20	29.37(3)	8.37
	970	1440	57.76(2)	8.20	60.775(93)	7.14	1448	51.77(8)	8.09	30.901(10)	8.06	1456	46(4)	8.07	29.48(3)	8.40
6.29	0	330	55.19(7)	7.82	58.856(25)	7.71	330	49.24(9)	7.97	30.267(11)	7.97	330	43.42(3)	7.68	28.87(5)	7.37
	400	654	55.35(4)	8.42	59.198(24)	8.39	661	49.48(10)	8.46	30.371(14)	8.62	669	43.62(3)	8.38	29.01(4)	8.20
	500	769	55.4(2)	8.64	59.35(19)	8.51	774	49.52(11)	8.66	30.406(13)	8.87	778	43.67(4)	8.63	29.04(4)	8.57
	600	883	55.49(2)	8.82	59.502(20)	8.64	887	49.54(11)	8.89	30.454(9)	9.01	892	43.71(4)	8.91	29.09(4)	8.84
	700	1003	55.54(3)	9.05	59.634(29)	8.93	1008	49.59(10)	9.11	30.489(6)	9.31	1012	43.78(5)	9.18	29.13(3)	9.22
	770	1093	55.56(2)	9.24	59.789(29)	8.89	1098	49.62(9)	9.29	30.524(11)	9.46	1103	43.79(5)	9.42	29.17(3)	9.47
	870	1227	55.74(2)	9.39	60.015(13)	8.87	1234	49.76(6)	9.44	30.584(9)	9.63	1240	44.05(4)	9.51	29.23(3)	9.70
	970	1377	55.94(2)	9.56	60.226(14)	9.09	1385	50(4)	9.54	30.681(12)	9.57	1392	44.29(6)	9.66	29.3(3)	9.97
	1070	1543	56.1(3)	9.80	60.513(9)	9.11	1553	50.23(11)	9.69	30.775(11)	9.64	1562	44.7(5)	9.70	29.39(4)	10.24
	1170	1733	56.56(2)	9.85	60.857(27)	9.13	1742	50.9(16)	9.48	30.898(7)	9.61	1750	45.27(6)	9.63	29.51(5)	10.33

* The unit cell volumes at room pressure and temperature conditions were obtained using Match! Software with FullProf from diffraction data obtained in the GNR Europe θ-θ benchtop diffractometer at P61B.
† Extrapolated V_0 for B2 structure (Köhler *et al.* 1997)
†† Extrapolated V_0 for B2 structure (Dewaele *et al.* 2012)
$ May have been heated by the x-ray beam
CsCl: 110,211,220,300
Pt: 111,200,220,311
KBr (B2): 110,200,210,211,310
Mo: 110,200,211,220,310
KCl (B2): 110,200,211
Re: 010,002,011,012,110,013,112,021

Table S4. For BT654, the calculated 'cross-calibrated' pressures and temperatures and their errors obtained using the EosCross app.

		**CsCl-Pt**						**KBr-Mo**						**KCl-Re**					
Load (MN)	Power (W)	T_t/c (K)	T_cross (K)	T error (K)	P_CsCl (GPa)	P_cross (GPa)	P error (GPa)	T_t/c (K)	T_cross (K)	T error (K)	P_KBr (GPa)	P_cross (GPa)	P error (GPa)	T_t/c (K)	T_cross (K)	T error (K)	P_KCl (GPa)	P_cross (GPa)	P error (GPa)
**1.09**	0	298.00	297.80	16.68	2.722	2.717	0.045	298.00	309.62	31.04	2.786	2.807	0.088	298.00	-	-	2.722	-	-
	100	391.83	401.35	17.50	2.706	2.728	0.052	395.50	413.71	47.78	2.672	2.712	0.118	399.17	308.72	115.60	2.735	2.488	0.332
	200	506.70	509.97	17.09	2.775	2.782	0.049	511.10	482.46	31.61	2.645	2.581	0.078	515.50	442.87	101.70	2.741	2.540	0.292
	300	633.62	646.26	14.60	2.775	2.804	0.043	637.85	559.54	29.71	2.530	2.356	0.074	642.08	551.53	80.42	2.662	2.411	0.249
	400	772.48	793.11	15.12	2.675	2.723	0.043	778.05	660.03	24.13	2.364	2.102	0.064	783.62	654.52	52.53	2.543	2.186	0.159
	500	925.22	941.92	21.04	2.546	2.585	0.056	931.45	770.10	26.20	2.256	1.898	0.072	937.68	831.32	38.75	2.453	2.160	0.136
	600	1091.70	1142.12	19.26	2.540	2.658	0.052	1097.10	897.10	23.48	2.172	1.728	0.065	1102.50	1087.24	48.13	2.455	2.413	0.166
	700	1268.55	1308.40	53.69	2.393	2.486	0.163	1274.05	1058.72	21.16	1.816	1.338	0.054	1279.55	1209.58	44.04	2.151	1.961	0.132
**2.9**	0	319.70	-	-	4.959	-	-	319.70	-	-	5.041	-	-	319.70	-	-	4.614	-	-
	100	387.72	359.40	21.38	5.226	5.160	0.066	391.15	-	-	5.294	-	-	394.58	355.81	140.52	4.957	4.850	0.489
	200	485.72	461.04	23.75	5.589	5.531	0.089	490.15	348.12	38.89	5.505	5.190	0.107	494.58	477.73	133.27	5.318	5.270	0.426
	300	594.63	581.25	24.59	5.732	5.701	0.093	598.70	471.48	37.26	5.596	5.313	0.103	602.77	601.79	144.70	5.437	5.433	0.431
	400	712.82	729.81	23.18	5.748	5.787	0.082	717.45	603.19	34.31	5.593	5.339	0.096	722.08	766.18	79.56	5.489	5.613	0.242
	500	839.75	836.64	41.01	5.662	5.654	0.112	844.05	788.98	61.52	5.693	5.571	0.221	848.35	873.96	102.42	5.505	5.577	0.302
	600	972.17	986.57	39.00	5.686	5.719	0.105	975.50	881.90	52.23	5.674	5.466	0.200	978.83	980.12	83.40	5.669	5.672	0.264
	700	1107.12	1149.65	26.78	5.703	5.802	0.073	1111.35	1009.16	47.42	5.640	5.413	0.177	1115.58	1085.06	68.64	5.724	5.638	0.228
	770	1215.85	1265.47	9.20	5.725	5.840	0.031	1220.75	1088.59	35.09	5.588	5.294	0.122	1225.65	1206.39	54.42	5.874	5.819	0.183
	870	1376.47	1467.47	72.32	5.759	5.971	0.175	1384.00	1267.66	29.08	5.389	5.131	0.072	1391.53	1349.64	48.81	5.714	5.596	0.164
**4.6**	0	323.50	323.94	38.37	6.224	6.224	0.157	323.50	412.41	111.94	6.541	6.738	0.335	323.50	370.01	137.68	6.018	6.146	0.389
	100	385.73	367.08	35.02	6.425	6.381	0.118	388.60	497.66	98.10	6.668	6.910	0.300	391.47	429.61	139.06	6.202	6.308	0.410
	200	472.67	466.38	33.58	6.645	6.630	0.108	476.60	532.57	82.25	6.815	6.939	0.256	480.53	506.25	122.48	6.443	6.516	0.353
	300	569.68	568.78	26.60	6.879	6.877	0.074	574.05	577.17	68.78	7.047	7.054	0.220	578.42	583.72	111.33	6.737	6.751	0.330
	400	675.22	680.88	31.75	7.159	7.172	0.107	680.25	652.40	63.76	7.288	7.226	0.202	685.28	672.71	106.72	7.051	7.015	0.323
	500	789.28	819.80	30.91	7.467	7.538	0.082	794.25	779.19	79.50	7.573	7.539	0.243	799.22	780.21	92.84	7.378	7.324	0.276
	600	908.77	944.66	23.18	7.713	7.796	0.060	914.10	884.62	69.48	7.820	7.754	0.213	919.43	915.47	89.17	7.702	7.691	0.266
	700	1037.73	1020.56	42.98	7.974	7.933	0.104	1043.60	985.84	60.71	8.040	7.912	0.185	1049.47	1049.82	84.53	8.012	8.013	0.268
	770	1136.85	1143.99	72.98	8.223	8.239	0.184	1141.95	1091.64	50.78	8.191	8.079	0.161	1147.05	1138.62	80.88	8.137	8.113	0.245
	870	1280.38	1368.71	75.50	8.352	8.558	0.181	1287.35	1266.52	47.22	8.204	8.157	0.146	1294.32	1259.84	83.29	8.203	8.104	0.275
	970	1439.88	1615.18	74.31	8.196	8.604	0.186	1448.05	1460.25	50.10	8.091	8.118	0.175	1456.22	1388.93	87.32	8.073	7.880	0.288
**6.29**	0	330.10	351.69	32.84	7.818	7.867	0.139	330.10	331.03	90.81	7.974	7.975	0.296	330.10	393.54	138.85	7.675	7.851	0.421
	400	653.68	659.41	25.28	8.418	8.431	0.093	661.05	601.86	86.47	8.458	8.326	0.298	668.42	705.61	122.73	8.377	8.484	0.389
	500	768.87	791.97	17.48	8.636	8.690	0.055	773.60	700.53	82.79	8.663	8.500	0.292	778.33	791.20	107.51	8.634	8.670	0.362
	600	882.40	915.36	19.60	8.817	8.893	0.069	887.20	846.04	67.10	8.892	8.800	0.259	892.00	906.35	107.07	8.907	8.948	0.363
	700	1002.85	1025.17	28.95	9.054	9.105	0.099	1007.55	938.36	53.56	9.110	8.956	0.220	1012.25	1003.72	91.37	9.177	9.152	0.329
	770	1093.10	1157.32	26.27	9.241	9.391	0.076	1097.90	1036.05	65.64	9.285	9.148	0.242	1102.70	1092.49	84.55	9.418	9.388	0.300
	870	1226.73	1320.30	12.61	9.390	9.609	0.046	1233.40	1170.79	49.75	9.444	9.305	0.175	1240.07	1201.72	76.48	9.513	9.402	0.265
	970	1377.33	1461.48	14.25	9.558	9.755	0.050	1384.80	1374.87	48.33	9.542	9.520	0.148	1392.27	1330.14	96.90	9.664	9.484	0.348
	1070	1542.53	1665.09	11.21	9.802	10.088	0.049	1552.40	1568.57	67.75	9.688	9.724	0.257	1562.27	1415.67	114.80	9.699	9.378	0.387
	1170	1732.52	1858.36	23.83	9.845	10.139	0.069	1741.35	1700.72	66.26	9.483	9.393	0.286	1750.18	1606.58	122.34	9.627	9.215	0.416

Table S5. Experimental conditions and unit cell volumes obtained by *in situ* X-ray diffraction for BT654 using only the same peaks**.**
Volumes are in Å^3, pressures in GPa. 2θ = 5.0298(6)°

Load (MN)	P (W)	T (K)	V_{CsCl}	P_{CsCl}	V_{Pt}	P_{Pt}	T (K)	$V_{KBr (B2)}$	$P_{KBr (B2)}$	V_{Mo}	P_{Mo}	T (K)	$V_{KCl (B2)}$	P_{KCl}	V_{Re}	P_{Re}
BenchXRD*:	-	300	70.0874	0	60.3793	0	300	62.93†	0	31.1723	0	300	54.5††	0	29.4280	0
ED-XRD:	-	300	70.11(2)	0	60.45(2)$	0	300	n/a	0	31.17(1)	0	300	n/a	0	29.45(4)	0
1.09	0	300	62.36(5)	2.72	59.83(45)	2.56	300	56.08(27)	2.66	30.838(15)	2.75	300	48.63(2)	2.75	29.2(7)	2.83
	100	392	62.855(76)	2.70	59.994(53)	2.48	396	56.49(9)	2.66	30.906(24)	2.63	399	49.02(4)	2.77	29.26(6)	2.91
	200	507	63.24(4)	2.79	60.155(52)	2.60	511	56.98(4)	2.67	30.962(18)	2.74	516	49.64(6)	2.72	29.32(4)	2.95
	300	634	63.89(4)	2.79	60.319(45)	2.82	638	57.98(10)	2.49	31.045(11)	2.70	642	50.44(9)	2.62	29.41(5)	2.93
	400	773	64.91(2)	2.70	60.589(36)	2.69	778	58.88(18)	2.41	31.136(14)	2.69	784	51.41(6)	2.53	29.5(6)	2.93
	500	925	66.29(6)	2.55	60.873(46)	2.64	932	60.19(7)	2.26	31.232(15)	2.73	938	52.54(11)	2.45	29.62(5)	2.71
	600	1092	67.52(3)	2.55	61.24(56)	2.40	1097	61.53(11)	2.18	31.335(14)	2.80	1103	53.7(14)	2.46	29.75(2)	2.48
	700	1269	67.75(29)	2.89	61.627(121)	2.23	1274	64.25(4)	1.83	31.505(15)	2.46	1280	56.2(4)	2.15	29.87(2)	2.53
2.90	0	320	58.66(8)	4.94	59.179(0)	5.94	320	52.48(15)	5.02	30.556(26)	5.29	320	45.97(9)	4.88	29.04(7)	5.13
	100	388	58.5(4)	5.21	59.228(7)	6.21	391	52.39(15)	5.25	30.561(21)	5.59	395	45.89(9)	5.17	29.08(7)	5.18
	200	486	58.31(11)	5.57	59.309(16)	6.53	490	52.36(15)	5.49	30.585(18)	5.85	495	46(7)	5.34	29.13(5)	5.23
	300	595	58.46(12)	5.72	59.447(26)	6.68	599	52.57(13)	5.57	30.637(18)	5.94	603	46.54(2)	5.44	29.2(5)	5.19
	400	713	58.84(9)	5.73	59.688(26)	6.41	718	52.91(11)	5.58	30.714(19)	5.87	722	46.94(3)	5.51	29.27(4)	5.24
	500	840	59.39(6)	5.65	59.956(27)	6.11	844	53.27(48)	5.60	30.797(19)	5.80	849	47.31(5)	5.53	29.35(4)	5.25
	600	972	59.84(6)	5.68	60.217(31)	5.92	976	53.7(50)	5.60	30.866(15)	5.91	979	47.31(5)	5.60	29.39(4)	5.70
	700	1107	60.31(4)	5.70	60.516(30)	5.63	1112	54.18(45)	5.58	30.949(14)	5.93	1116	47.69(8)	5.70	29.46(3)	5.89
	770	1216	60.69(3)	5.74	60.76(38)	5.42	1221	54.63(31)	5.54	31.019(14)	5.92	1226	48.12(20)	5.69	29.53(3)	5.96
	870	1377	61.31(3)	5.76	61.045(51)	5.48	1384	55.37(48)	5.46	31.154(18)	5.70	1392	48.93(12)	5.61	29.65(2)	5.91
4.60	0	324	56.97(13)	6.22	58.974(58)	7.04	324	50.83(17)	6.40	30.451(48)	6.25	324	44.83(2)	6.02	28.99(7)	5.81
	100	386	56.89(7)	6.43	59.009(59)	7.32	389	50.88(18)	6.50	30.476(43)	6.33	392	44.84(4)	6.20	29.01(6)	6.00
	200	473	56.87(6)	6.65	59.112(51)	7.45	477	50.93(17)	6.66	30.494(40)	6.59	481	44.84(1)	6.45	29.04(6)	6.31
	300	570	56.86(2)	6.88	59.224(42)	7.61	574	50.91(17)	6.88	30.506(31)	6.96	579	44.83(2)	6.73	29.07(5)	6.69
	400	675	56.82(6)	7.16	59.352(82)	7.77	680	50.88(15)	7.14	30.528(30)	7.30	685	44.82(3)	7.05	29.1(5)	7.07
	500	789	56.82(8)	7.42	59.507(98)	7.88	794	50.89(15)	7.39	30.562(35)	7.58	799	44.82(2)	7.38	29.14(5)	7.45
	600	909	56.86(9)	7.66	59.683(88)	7.96	914	50.91(5)	7.64	30.598(29)	7.88	920	44.84(2)	7.70	29.19(4)	7.75
	700	1038	56.86(1)	7.97	59.877(81)	8.04	1044	50.89(12)	7.94	30.643(28)	8.15	1050	44.9(4)	8.01	29.25(4)	8.05
	770	1137	56.83(2)	8.22	60.004(118)	8.21	1142	50.97(12)	8.10	30.681(20)	8.32	1147	45.04(2)	8.13	29.29(4)	8.20
	870	1281	57.09(1)	8.35	60.343(97)	7.79	1288	51.31(13)	8.12	30.782(20)	8.22	1294	45.39(6)	8.20	29.37(4)	8.39
	970	1440	57.76(4)	8.19	60.688(33)	7.51	1448	51.92(19)	7.96	30.905(18)	8.02	1456	46(7)	8.07	29.47(4)	8.43
6.29	0	330	55.24(11)	7.76	58.768(35)	8.17	330	49.24(14)	7.68	30.267(20)	7.98	330	43.42(5)	7.67	28.87(6)	7.36
	400	654	55.34(6)	8.42	59.122(21)	8.78	661	49.48(16)	8.39	30.356(16)	8.75	669	43.63(5)	8.37	29.01(6)	8.18
	500	769	55.38(3)	8.66	59.278(8)	8.87	774	49.52(17)	8.59	30.397(17)	8.95	778	43.67(7)	8.63	29.04(5)	8.60
	600	883	55.44(2)	8.86	59.435(8)	8.97	887	49.54(17)	8.82	30.448(22)	9.06	892	43.72(7)	8.90	29.09(5)	8.84
	700	1003	55.49(3)	9.10	59.612(17)	9.04	1008	49.59(16)	9.11	30.484(18)	9.35	1012	43.78(9)	9.17	29.13(5)	9.20
	770	1093	55.56(4)	9.24	59.769(18)	8.98	1098	49.62(15)	9.28	30.528(19)	9.43	1103	43.8(7)	9.42	29.16(4)	9.48
	870	1227	55.75(5)	9.38	60.022(25)	8.84	1234	49.76(10)	9.44	30.589(18)	9.58	1240	44.05(6)	9.51	29.23(4)	9.70
	970	1377	55.98(5)	9.52	60.229(22)	9.07	1385	50(7)	9.54	30.679(23)	9.59	1392	44.29(10)	9.66	29.3(4)	10.02
	1070	1543	56.13(8)	9.77	60.526(26)	9.05	1553	50.23(17)	9.69	30.779(22)	9.61	1562	44.7(8)	9.69	29.38(7)	10.32
	1170	1733	56.59(5)	9.82	60.861(36)	9.11	1742	50.9(28)	9.49	30.899(17)	9.60	1750	45.27(10)	9.63	29.5(8)	10.40

* The unit cell volumes at room pressure and temperature conditions were obtained using Match! Software with FullProf from diffraction data obtained in the GNR Europe θ-θ benchtop diffractometer at P61B.
† Extrapolated V_0 for B2 structure (Köhler *et al.* 1997)
†† Extrapolated V_0 for B2 structure (Dewaele *et al.* 2012)
$ May have been heated by the x-ray beam.
All following peaks for each material were exclusively used in the fitting of all diffraction patterns:
CsCl: 110,211,221 Pt: 111,200
KBr (B2): 110,200,211 Mo: 110,211,220,310
KCl (B2): 110,200,211 Re: 010,002,011,012,110,013,112

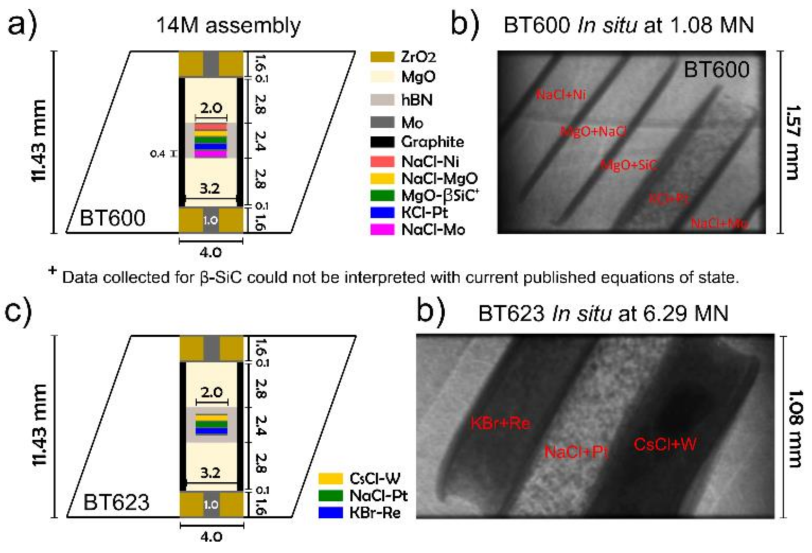

Figure S1

Cell assembly designs and X-ray radiograph images of the two experiments without thermocouple BT600 (a,b) and BT623 (c,d). The pairs of PT calibrants are colour-coded (see other suppl. figures). MgO-βSiC is ignored in this study. The βSiC diffraction data could not be interpreted, as no published equation of state offered meaningful results. The samples are separated by 0.05 mm Mo discs.

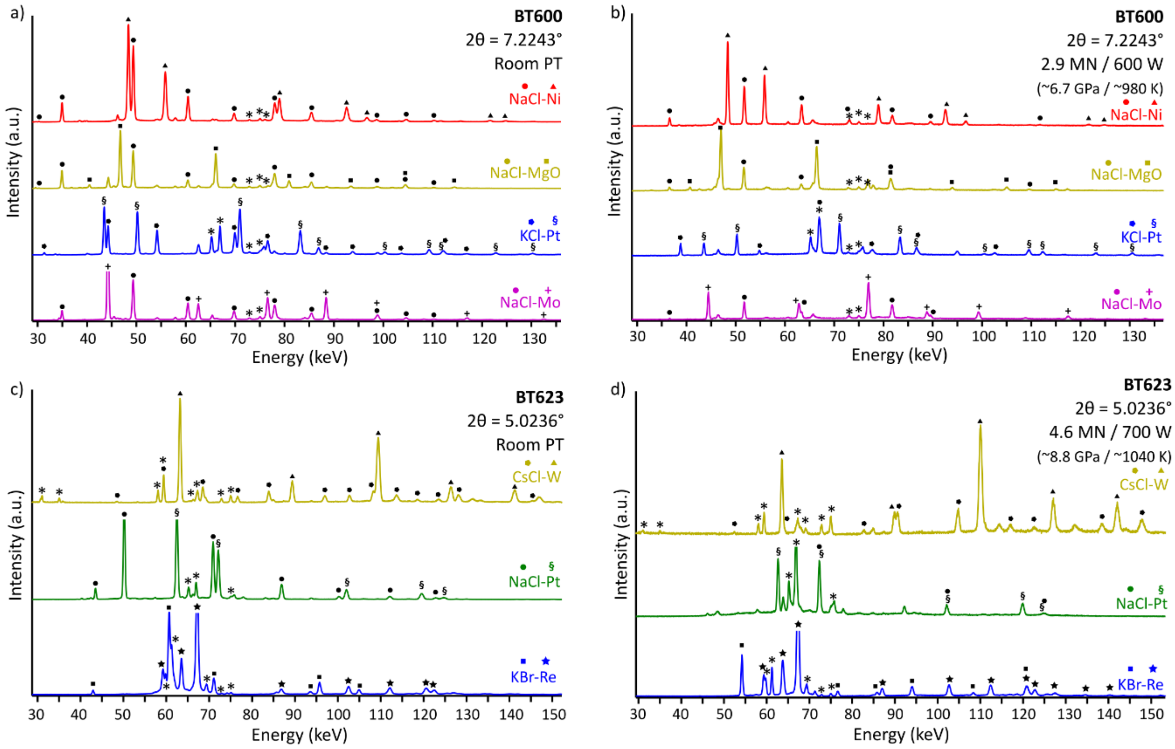

Figure S2

Representative energy-dispersive XRD data of the two experiments without thermocouple BT600 (a,b) and BT623 (c,d). In most cases, all peaks are accounted for, except where no symbol is given. Fluorescence lines, including those from the Pb detector shielding, are indicated by asterisks. The detector position and experimental conditions are indicated in each panel.

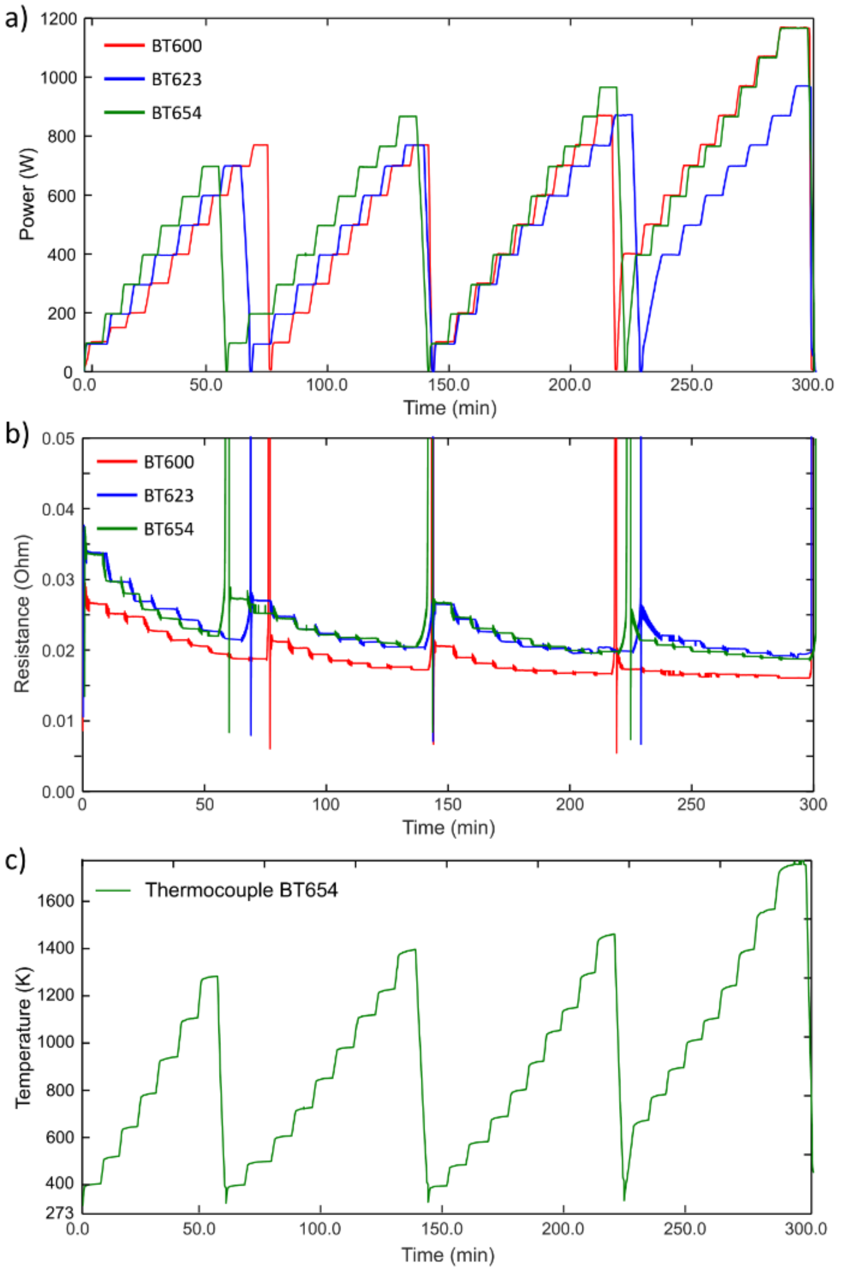

Figure S3

Heating data of the three experiments plotted against time as reported by the 10kW DC power supply. (a) As shown, 4 cycles of heating (at 4 different press load increments) were performed for BT600, BT623, and BT654. At each cycle, the same heating steps were adhered to 0, 100, 200, 300, 400, 500, 600, 700, 770, 870, 970, 1070, 1170 W. The higher power steps were not attempted at lower press load, or some samples would melt due to too high temperatures. (b) Resistance of the furnaces, showing BT623 and BT654 have identical resistances throughout the experiment, and BT600 somewhat lower resistance, likely from a better electrode – anvil contact. (c) The reported thermocouple temperature of BT654, faithfully reproducing heating step over the 4 heating cycles. At each step, at constant power, temperature crept upwards by a few degrees. So, there is a small temperature difference between the time at each step the first XRD pattern was collected and the last XRD pattern was collected. This was accounted for in the pressure calculations.

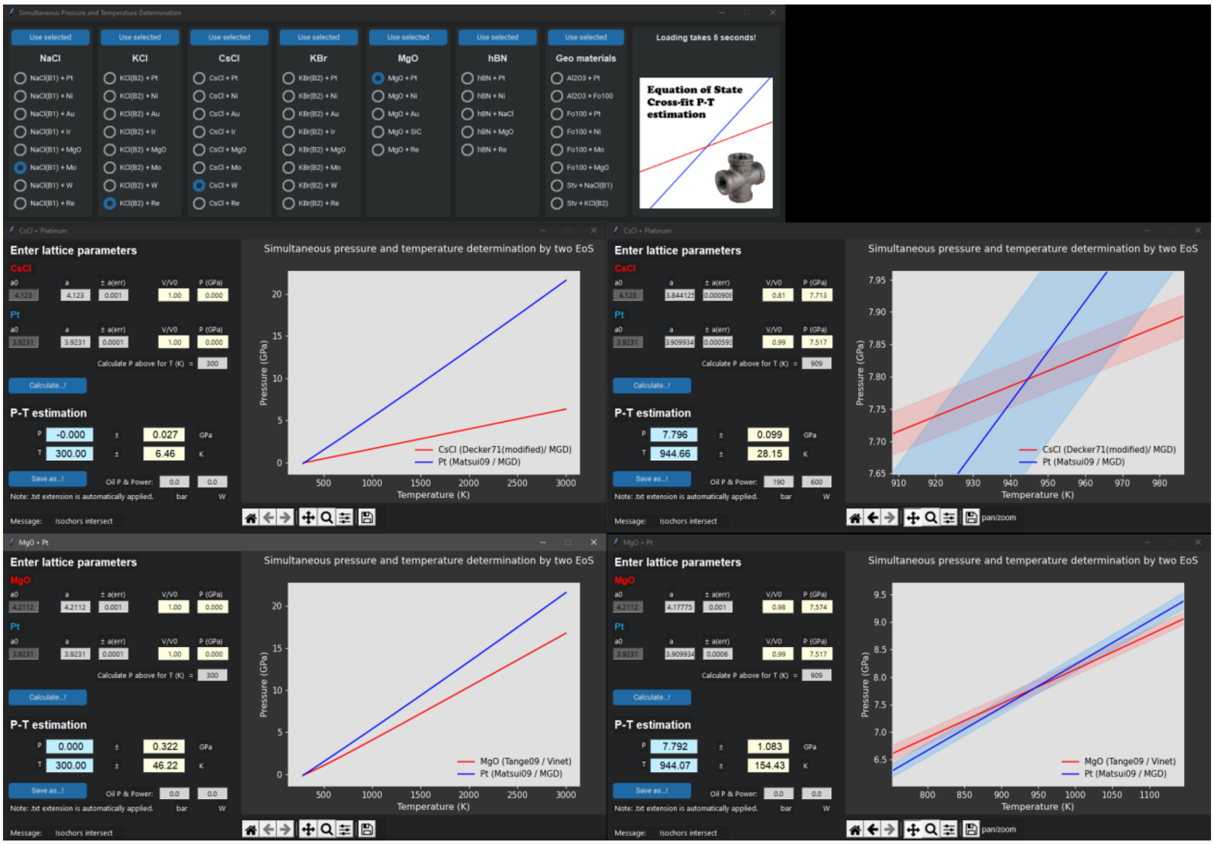

Figure S4

The interfaces of the custom-written software EosCross (first introduced in Farla *et al.* (2022)). The python software consists of a main menu (top) window, presenting many difference choices of PT calibrant pairs for the user at the beamline. The first combination, CsCl + Pt, is evaluated in this study and the joint PT estimation offers the lowest errors (0.027 GPa and 6.5 K) for given errors in the lattice parameters due to two strongly different isochor slopes. The second combination, MgO + Pt, is an example of a poor choice where the isochor slopes are nearly parallel. Even with small errors in the lattice parameters (as given), the overall errors of the joint PT estimation can be very large (0.322 GPa & 46.2 K). On the right, at 190 bar and 600 W heating power, showing the results of a joint PT estimation for CsCl and Pt, and a hypothetical case for MgO yielding the same P and T. The errors of the cross-calibration are visualised by the intersection of the light-coloured isochors in the lower-left and top-right in the plots. The light-coloured iscochors are plotted from the same EOS calculations, but with a positive and negative contribution of the error in the lattice parameters.

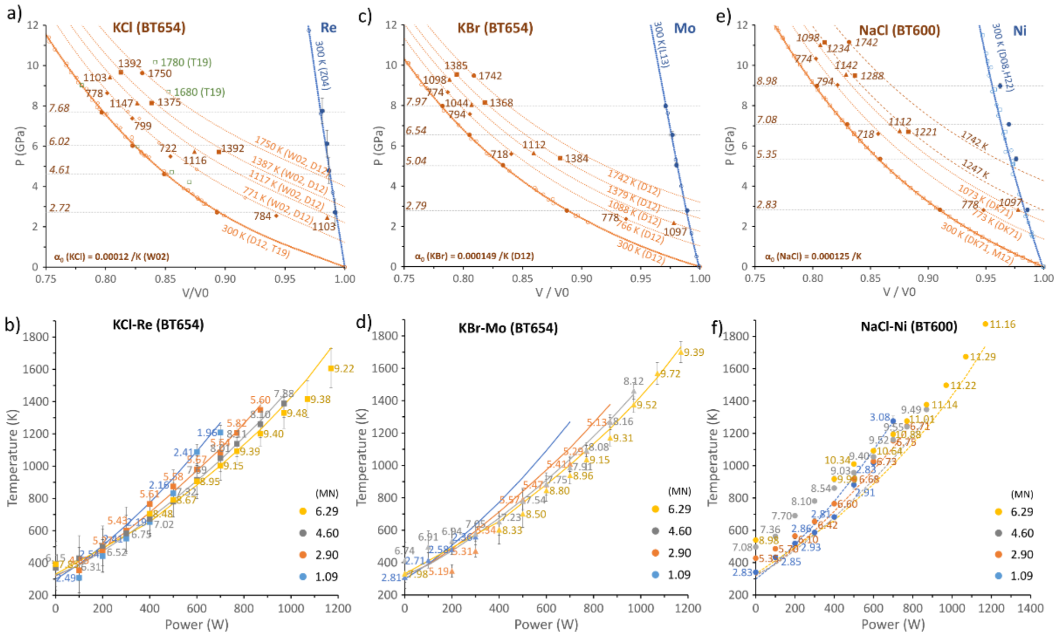

Figure S5

(a, c) P – V/V_0 diagrams calculated for KCl and KBr (BT654) using the thermocouple temperatures and (e) for NaCl (BT600) using the power-temperature relationship of BT654. Various curves (light orange, blue) and data points (open symbols) from previous studies are included and referenced in brackets. W02: (Walker *et al.*, 2002), T19: (Tateno *et al.*, 2019), Z04: (Zha *et al.*, 2004), D12: (Dewaele *et al.*, 2012), L13: (Litasov *et al.*, 2013), DK71: (Decker, 1971), M12: (Matsui *et al.*, 2012), D08: (Dewaele *et al.*, 2008), H22: (Hirao *et al.*, 2022).
(b, d, f) Power-Temperature curves (solid lines) from a thermocouple and dashed lines from the power-temperate relationship of BT654. Data points represent joint calculations of P (in labels) and T using various equations of state of each material (see Table 1) and the measured unit cell volumes at given press load and heating power.

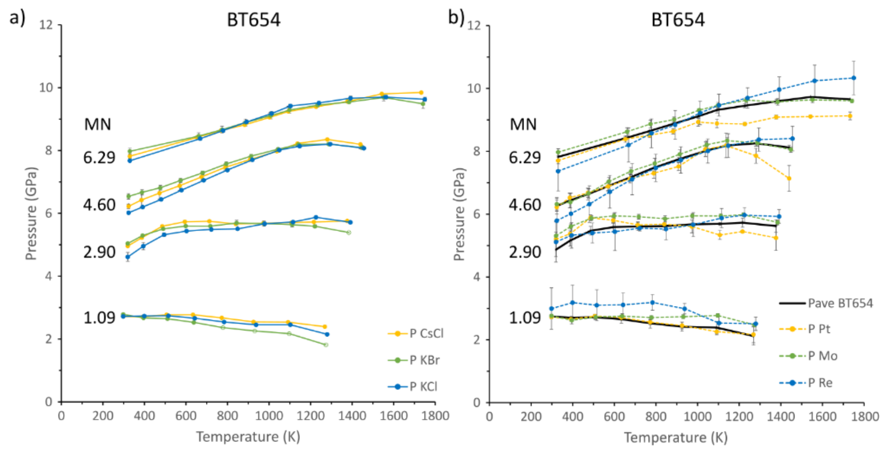

Figure S6

Calculated pressures of the PT calibrant pairs for BT654. (a) Pressures calculated for the halides based on the thermocouple temperatures (error bars are very small, often not larger than the symbol size). (b) Pressures calculated for the metals based on the thermocouple temperatures. The thick black curves are averages of the halide pressures in (a), for comparison.

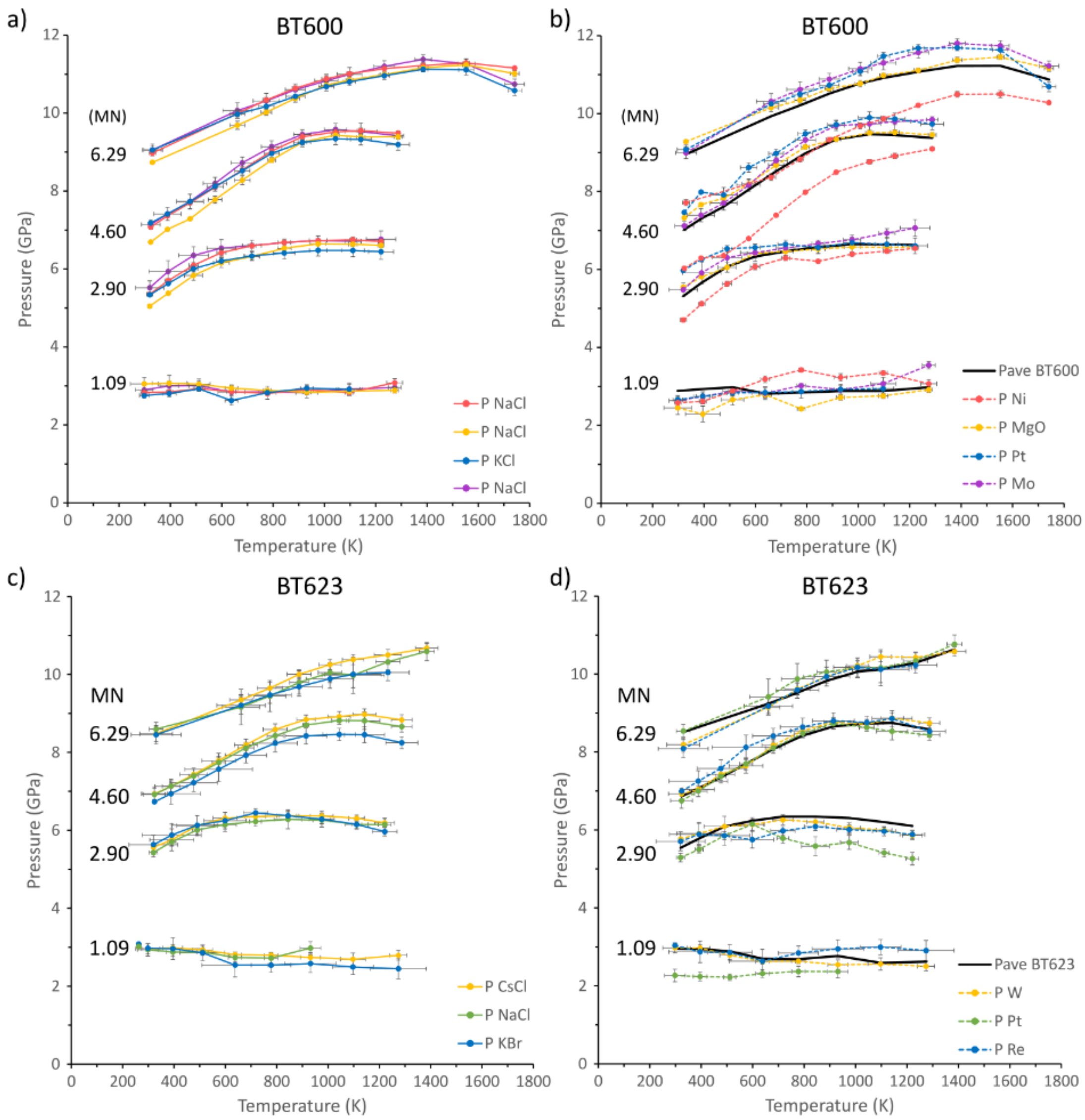

Figure S7

Calculated pressures of the PT calibrant pairs for BT600 and BT623. (a) Pressures calculated for the halides (a) and (b) metals using the power-thermocouple relationship of BT654. The thick black curves are averages of the halide pressures in (a), for comparison. Note, the highest pressures were achieved in BT600 at the same press loads as in the other experiments. This is because the TF08 Fujilloy anvils were brand new for BT600, reused for BT623, and finally for BT654. This demonstrates how plastic deformation of WC at the truncated edges (corners) of the anvils degrades pressure efficiency generation after multiple uses. This effect is often (unintentionally) ignored in offline multi anvil experiments, with consequence of potential serious errors.

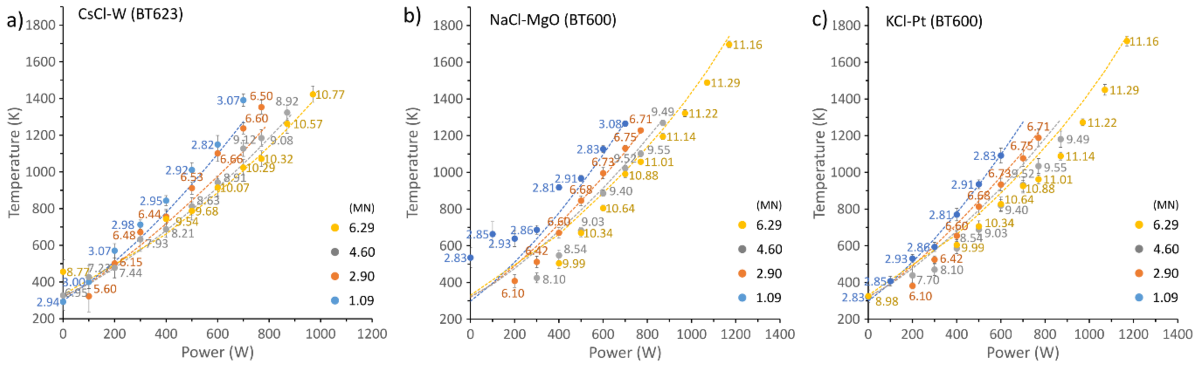

Figure S8

Additional jointly calculated PT data for BT600 and BT623 for calibrants used in the assemblies. The power-temperature curves (dashed lines) are based on the power-temperate relationship of BT654. Data points represent joint calculations of P (in labels) and T using various equations of state of each material (see Table 1) and the measured unit cell volumes at given press load and heating power. In many cases, the agreement of the joint PT calculations (data points) agree quite well with the temperature curves of BT654, suggesting the (nearly) identical cell assemblies indeed faithfully reproduced temperatures at each press load step.

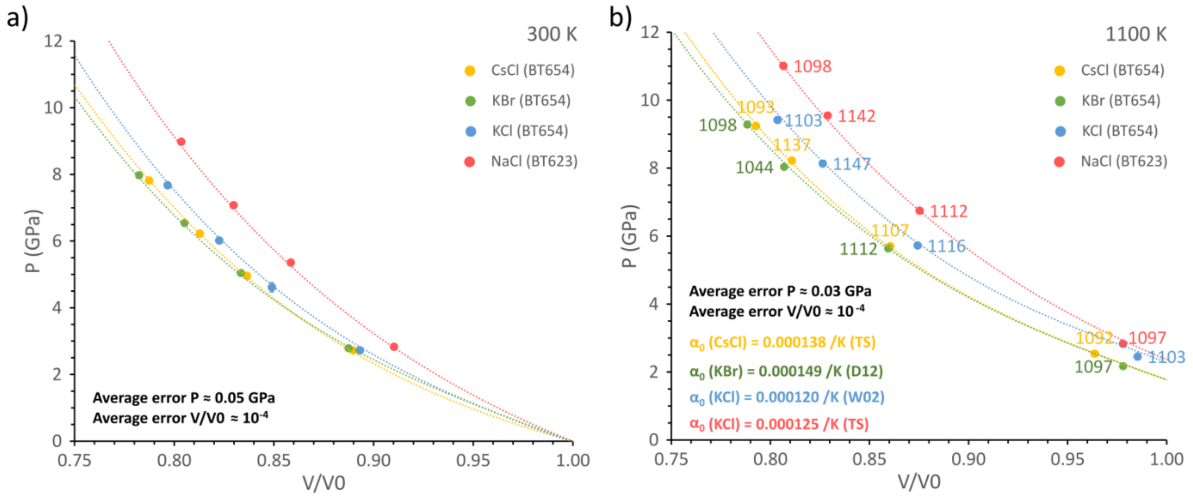

Figure S9

Comparison of the compressibility of the halides used in this study (CsCl, KBr, KCl, and NaCl) at room temperature (a) and around 1100 K (b). The dotted curves are obtained from their respective equations of state (Table 1). The thermal expansion (α) of each phase is as given, TS: ‘This Study’, D12: (Dewaele *et al.*, 2012), W02: (Walker *et al.*, 2002). It is clear that NaCl, followed by KCl, are less compressible and exhibit larger thermal pressures than CsCl and KBr.

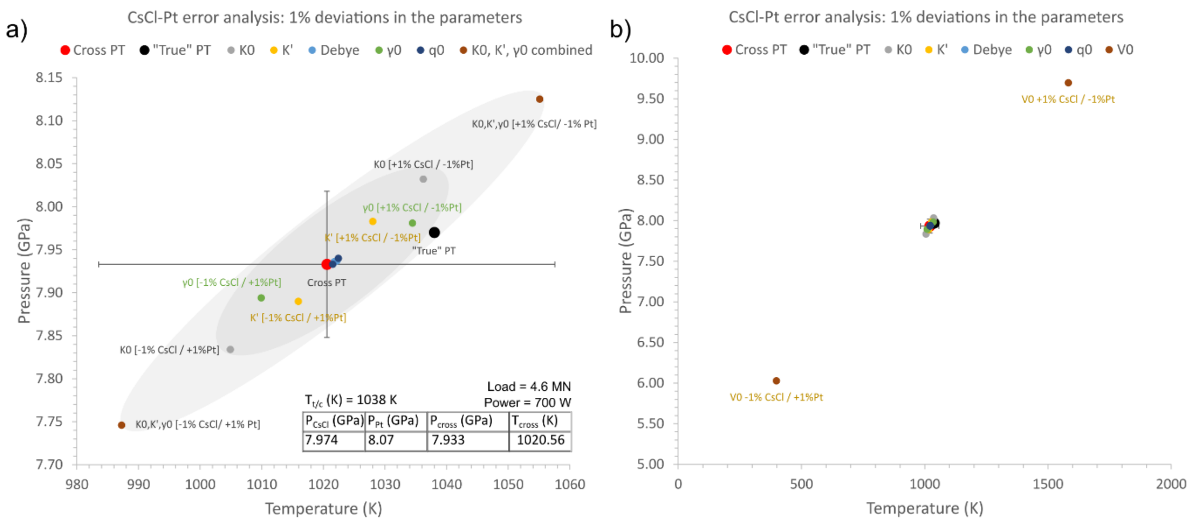

Figure S10

Calculations of joint P,T with ±1% variations in the EoS parameters. The error bars on the Cross PT (red data point) are ± 0.087 GPa and ± 37K. Only ±1% differences in K_0 results in P,T estimations outside this range, as well as when differences of -1% in CsCl and +1% in Pt and vice versa are combined for K_0, K' and γ_0.

References

Decker, D. L. (1971). *Journal of Applied Physics* **42**, 3239–3244.

Dewaele, A., Belonoshko, A. B., Garbarino, G., Occelli, F., Bouvier, P., Hanfland, M. & Mezouar, M. (2012). *Phys. Rev. B* **85**, 214105.

Dewaele, A., Torrent, M., Loubeyre, P. & Mezouar, M. (2008). *Phys. Rev. B* **78**, 104102.

Farla, R., Bhat, S., Sonntag, S., Chanyshev, A., Ma, S., Ishii, T., Liu, Z., Néri, A., Nishiyama, N., Faria, G. A., Wroblewski, T., Schulte-Schrepping, H., Drube, W., Seeck, O. & Katsura, T. (2022). *J Synchrotron Rad* **29**, 409–423.

Hirao, N., Akahama, Y. & Ohishi, Y. (2022). *Matter and Radiation at Extremes* **7**, 038403.

Litasov, K. D., Dorogokupets, P. I., Ohtani, E., Fei, Y., Shatskiy, A., Sharygin, I. S., Gavryushkin, P. N., Rashchenko, S. V., Seryotkin, Y. V., Higo, Y., Funakoshi, K., Chanyshev, A. D. & Lobanov, S. S. (2013). *Journal of Applied Physics* **113**, 093507.

Matsui, M., Higo, Y., Okamoto, Y., Irifune, T. & Funakoshi, K.-I. (2012). *American Mineralogist* **97**, 1670–1675.

Tateno, S., Komabayashi, T., Hirose, K., Hirao, N. & Ohishi, Y. (2019). *American Mineralogist* **104**, 718–723.

Walker, D., Cranswick, L. M. D., Verma, P. K., Clark, S. M. & Buhre, S. (2002). *American Mineralogist* **87**, 805–812.

Zha, C.-S., Bassett, W. A. & Shim, S.-H. (2004). *Review of Scientific Instruments* **75**, 2409–2418.
